# Emission factors for polycyclic aromatic hydrocarbons from laboratory biomass-burning and their chemical transformations during aging in an oxidation flow reactor

**DOI:** 10.1016/j.scitotenv.2023.161857

**Published:** 2023-01-30

**Authors:** Deep Sengupta, Vera Samburova, Chiranjivi Bhattarai, Hans Moosmüller, Andrey Khlystov

**Affiliations:** a Desert Research Institute, Reno, NV, USA; b University of California, Berkeley, CA, USA

**Keywords:** Biomass burning, Polycyclic aromatic hydrocarbons, Oxidation flow reactor, Particle phase, Gas phase, Toxic equivalency factor

## Abstract

Atmospheric polycyclic aromatic hydrocarbons (PAHs) can be emitted from different combustion sources including domestic biomass burning, internal combustion engines, and biomass burning (BB) in wild, prescribed, and agricultural fires. With climate warming and consequent global increases in frequency and severity of wildfires, BB is a dominant source of PAHs emitted into the atmosphere.

In this study, six globally and regionally important and representative fuels (Alaskan peat, Moscow peat, Pskov peat, eucalyptus, Malaysian peat, and Malaysian agricultural peat) were burned under controlled conditions in the combustion chamber facility at the Desert Research Institute (DRI, Reno, NV, USA). Gas- and particle-phase BB emissions were aged in an oxidation flow reactor (OFR) to mimic five to sevendays of atmospheric aging. To sample gas- and particle-phase BB emissions, fresh and OFR-aged biomass-burning aerosols were collected on Teflon-impregnated glass fiber filters (TIGF) in tandem with XAD resin media for organic carbon speciation. The objectives of this study were to i) quantify the emission factors for 113 PAHs emitted from the combustion of the six selected fuels, ii) characterize the distribution of PAH compounds between gas and particle phases for these fuels, iii) identify the changes in PAHs during OFR-aging, and iv) evaluate toxicity potential with characterized compounds.

We found that combustion emissions of gas-phase PAHs were more abundant (>80 % by mass) than particle-phase PAHs, for emissions from all combusted fuels. The mass fraction of substituted napthalenes in Moscow peat and Malaysian peat emissions were ~70 % & 84 %, respectively, whereas in Eucalyptus the same fraction was <50 %, which indicates that these substituted compounds can be used as tracers for peat emissions. Mass concentrations of gas- and particle-phase PAHs were reduced by ~70 % after OFR oxidation. However, the understanding of the fate of PAHs during OFR oxidation requires further investigations. Our results also indicate that the PAH toxicity of BB samples would be underestimated by 10–100 times if only the BaP_eq_ for the 16 US EPA priority PAHs in the particle phase are included.

## Graphical Abstract

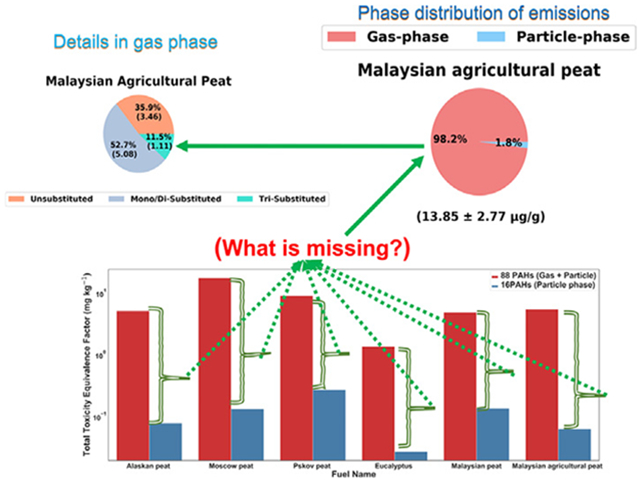

## 1. Introduction

Biomass Burning (BB) contributes significantly to particulate matter (PM) and trace gas concentrations in the atmosphere (Andreae and Merlet, 2001; Andreae, 2019). BB can cause short- and long-term ecological (Stephens et al., 2014) and climatic impacts (Jolly et al., 2015; Liu et al., 2014), disturbing the equilibrium of climate-vegetation-fire interactions (Harris et al., 2016). BB emissions are often responsible for air quality degradation on regional (Matteo et al., 2019) and global scales (Chen et al., 2017; Vakkari et al., 2018). BB smoke contains numerous hazardous air pollutants and it is of the essence to evaluate population health effects from BB smoke exposure (Reid et al., 2016). With increased frequency and size of BB events (Holden et al., 2018; Westerling et al., 2011) and projected anthropogenically induced fire activity in the 21st century (Hurteau et al., 2014), it is important to understand the chemical composition of BB emissions, estimate and/or project their potential health impacts (Cançado et al., 2006; Jaffe et al., 2004; Saffari et al., 2015; Shrivastava et al., 2017; Sigsgaard et al., 2015), and consequently relate those to mortality (Johnston et al., 2012).

Polycyclic Aromatic Hydrocarbons (PAHs), mainly due to their carcinogenic and mutagenic potential (Atkinson and Arey, 1994; Shen et al., 2014), have received special attention amongst other compound classes in organic aerosols derived from combustion processes. PAHs are found ubiquitously in the atmosphere and their most common sources are domestic burning (Du et al., 2021; Kim Oanh et al., 2002; Shen et al., 2012), fossil fuel combustion (Ali et al., 2021; Geng et al., 2014; Liu et al., 2008), agricultural waste burning (ChooChuay et al., 2022; Dhammapala et al., 2007; Shen et al., 2011), and wildland fires (Campos et al., 2019; Chen et al., 2018; Vachula et al., 2022; Wentworth et al., 2018), with a substantial increase in projected emissions by 2030 (Shen et al., 2013). PAH concentrations in BB emissions and the relative abundance of PAH compounds in BB emissions from different fuels are controlled by fuel composition as well as by combustion conditions (Jenkins et al., 1996), including relative humidity (Yuan et al., 2008), fuel moisture (McMohan and Tsoukalas, 1987), and combustion temperature (Faccinetto et al., 2011; Stein and Fahr, 1985). The influence of large BB events on urban atmospheres is characterized by PAH concentrations as has been demonstrated by studies in Europe (Mandalakis et al., 2005) and Asia (He et al., 2010). Source apportionment studies in Korea (Kim et al., 2013), India (Rajput et al., 2011), and China (Wang et al., 2014) also presented the contribution of different BB sources to total atmospheric PAH concentrations. As PAH emissions vary widely between fuels (Samburova et al., 2016; Shen et al., 2017) and combustion conditions, laboratory studies can be designed to perform source characterization (e.g., Akagi et al., 2011) under controlled conditions to develop better source profiles for pollutants, including PAHs. Such data are essential for atmospheric modeling (Friedman and Selin, 2012; Zhang et al., 2017) and adequate assessment of effects of BB emissions on human health and the environment. Studies on source characterization of PAHs are sparse and often restricted to the 16 U.S. EPA (United States Environmental Protection Agency) prioritized PAHs (Dong et al., 2020; Samburova et al., 2017), with a focus on particle-phase PAHs. However, PAHs vary widely in their volatility (depending on the number of aromatic rings in their structure) and thus can exist in both gas and particle phase. The phase partitioning of PAHs can control their fate during atmospheric transport, as well as their potential toxicity (Sofowote et al., 2010; Wei et al., 2015). To develop better understanding of PAH source profiles, Samburova et al. (2016) performed a detailed chemical characterization of both gas- and particle-phase PAHs in fresh BB emissions from fuels found in high latitude peatlands and in boreal and semi-arid regions of the U.S.. It was found that the analyzed 113 PAHs contributed very little (up to 0.16 %) to BB brown carbon absorptivity and that the 16 EPA-prioritized PAHs contribute only about a quarter of gas- and particle-phase PAH mass.

The total PM_2.5_ attributable number of premature deaths was ~3.2 million in 2010 globally and is forecasted to increase over time, especially in low income countries (Apte et al., 2015). BB emissions can lead to both acute and chronic exposure to hazardous air pollutants, including PAHs, for populations in the proximity of large wildfire impacted areas (O’Dell et al., 2020; Wentworth et al., 2018). Even though the PAH mass fraction in BB emissions is smaller than that of other chemical classes of compounds (e.g., sugars, methoxyphenols) these PAHs can contribute significantly towards overall oxidative potential (Daellenbach et al., 2020) and cytotoxicity (Niu et al., 2020; Sun et al., 2018).

Source apportionment studies demonstrated that some PAHs can survive long range atmospheric transport (Friedman et al., 2014; Sofowote et al., 2011), at the same time, chemical transformation of PAHs during transport may produce nitro- and/or oxo-PAHs (Kojima et al., 2010; Reisen and Arey, 2005). Previous studies have shown that nitrated and oxygenated PAHs are potentially more toxic than their parent compounds (Bandowe and Meusel, 2017; Mesquita et al., 2014). Laboratory studies with surrogate PAH compounds showed that oxidation of PAHs can occur in gas phase (Zhou and Wenger, 2013) and also can be driven by particle bound heterogeneous oxidations (Liu et al., 2012; Zhou et al., 2019). In addition, the role and contribution of anthropogenic PAHs to biogenic Secondary Organic Aerosol (SOA) formation has been quantified with chamber based experiments (Zelenyuk et al., 2017).

Recently, Oxidation Flow Reactors (OFRs) have been used in conjunction with laboratory combustion experiments to characterize potential aging occurring during transport of BB emissions (Fortenberry et al., 2018; Sengupta et al., 2020). However, present knowledge of chemical transformation for a complex mixture of hundreds of BB compounds, including PAHs, during atmospheric transport is still very limited. In this study, we performed BB experiments with six globally and regionally important wildland biomass fuels (i.e., Alaskan peat, Moscow peat, Pskov peat, Eucalyptus, Malaysian peat, and Malaysian agricultural peat) (Watts et al., 2020) in a combustion chamber with volume of 9 m^3^. BB emissions sampled from this chamber were subjected to OFR oxidation to mimic five to seven days of atmospheric aging. Gas- and particle-phase PAHs of both fresh and OFR-aged BB emissions were collected, extracted, and quantitatively analyzed for 113 individual PAHs. Their emission factors (EF) as well as Toxic Equivalency factors (TEFs) were calculated for all collected samples to assess the difference between PAH-toxicity of fresh and OFR-aged BB emissions.

## 2. Experimental

### 2.1. Reagents and materials

Deuterated PAH standards were purchased from Sigma-Aldrich (St. Louis, MO, USA). Unsubstituted PAHs and substituted (alkylated) PAHs were purchased from AccuStandard (New Haven, CT, USA) and Cambridge Isotope Laboratories, Inc. (Andover, MA, USA) respectively. High-performance liquid chromatography (HPLC) grade toluene, acetone, and dichloromethane were obtained from Fisher Scientific (Fair Lawn, NJ, USA). PM was collected on pre-fired 47-mm diameter Teflon-impregnated glass fiber (TIGF) filters (47-mm in diameter, Fiber Film T60A20, Pall Life Sciences, Ann Arbor, MI, USA) for organic analysis.

### 2.2. Fuel selection and biomass-burning experimental set-up

We selected six globally and regionally important BB fuels: Alaskan peat, Moscow peat, Pskov peat, Eucalyptus, Malaysian peat, and Malaysian agricultural peat (Watts et al., 2020). Five of these were peat fuels from different geographical locations, representing smoldering combustion, and one (Eucalyptus) representing flaming combustion. A more detailed description of these fuels and criteria for their selection has been given by Sengupta et al. (2020).

BB experiments were conducted using DRI's BB facility for combustion of the selected fuels under controlled conditions. A close replicate of this facility was described previously (Tian et al., 2015), and a detailed description of the experimental setup was presented elsewhere (Bhattarai et al., 2018; Sengupta et al., 2020). The duration of smoldering combustion experiments ranged from 69 to 255 min (fuel weight = 100 - 200 g), whereas the average duration of flaming combustion experiments was 50 min (fuel weight = 1 kg) (During all experiments, both fresh (sampled directly from the combustion chamber) and aged (oxidized in the OFR) emissions were continuously collected at room temperature (25 ± 1 °C) on TIGF filters (for particle phase), followed by XAD cartridge sampling (for gas phase), for detailed chemical speciation. Different levels of oxidation inside OFR were achieved by varying lamp (both 185 nm and 254 nm) voltages and calibrating against carbon monoxide. We set the lamp voltage to 5–7 days of equivalent atmospheric aging and performed aging experiments. The online instruments alternated every 10 min between sampling fresh and OFR-aged emissions using a computer-controlled valve system. We employed a bypass flow to keep the flow from the BB chamber and through the OFR constant when online instruments switched between sampling fresh and aged emissions. To protect online instruments from high ozone concentrations produced in the OFR, ozone scrubbers which by itself can remove some particles were installed in front of the instruments’ inlets. The ozone scrubbers were loaded with charcoal, followed by Carulite 200 catalyst (Carus Corp., Peru, IL, USA). There were no ozone scrubbers before the filter-XAD setup, which could cause further oxidation of organic compounds on filter surfaces during sampling. The reaction rates between organics and ozone, however, are orders of magnitude lower than those of OH oxidation reactions (Finlayson-Pitts and Pitts Jr., 1999). Therefore, we assume that reactions with OH radicals were primarily responsible for changes in organic compounds associated with fresh gas- and particle-phase emissions. All samples were stored at −20 °C for 2–3 days before extraction.

### 2.3. Sample preparation and GC–MS analysis

Collected 47-mm diameter TIGF filters and XAD samples were spiked with deuterated internal PAH standards and extracted separately using an accelerated solvent extractor (ASE) instrument (DIONEX, ASE-300, Salt Lake City, UT, USA). Naphthalene-*d*_8_, biphenyl-*d*_10_, acenaphthene-*d*_10_, phenanthrene-*d*_10_, anthracene-*d*_10_, pyrene-*d*_12_, benz(*a*)anthracene-*d*_12_, chrysene-*d*_12_, benzo(*k*)fluoranthene-*d*_12_, benzo(*e*)pyrene-*d*_12_, benzo(*a*)pyrene-*d*_12_, perylene-*d*_12_, benzo(*ghi*)perylene-*d*_12_, and coronene-*d*_12_ were used as internal standards. The ASE extraction parameters were temperature: 80 °C, solvents: dichloromethane followed by acetone (150 mL each), pressure: 10.3 MPa, and extraction time: 15 min. After extraction, the volume of extract solution was reduced to 1 mL with a rotary evaporator (Rotavapor R-124, BÜCHI, New Castle, USA) under a gentle vacuum at 35 °C, filtered with a 0.2-μm pore-size polytetrafluoroethylene membrane filter (Whatman, Florham Park, NJ, USA), and transferred into a 2-mL volume amber glass vial. Half of the extract (0.5 mL) was prepared for further GC–MS analysis of PAHs (Samburova et al., 2016) using solid-phase extraction (SPE) procedure. SPE was used to separate the PAH-fraction from polar species that can contribute to high matrix background. For this purpose, solvent of the PAH-fraction (acetone-dichloromethane) was exchanged on hexane. NH2-SPE cartridges (Sep-Pak^®^ Vic 3 cc, 500 mg, Waters Corporation, Milford, MA, USA) were preconditioned with 10 mL of dichloromethane followed by 10 mL of hexane. The extract was run through the conditioned NH2-SPE cartridge and PAHs were eluted with hexane/dichloromethane (98/2; v/v), followed by 10 mL of hexane/dichloromethane (80/20; v/v). The PAH-fraction was pre-concentrated under ultra-high purity N_2_-stream and the solvent was exchanged on toluene and pre-concentrated, again under N_2_-stream, to 0.5 mL volume.

BB sample extracts were analyzed with electron impact gas chromatography mass spectrometry (GC–MS). A Scion-456 GC, equipped with a CP-8400 autosampler and interfaced to EVOQ-TQ triple quadrupole Mass Spectrometer (Bruker, Billerica, MA, USA), was used to perform splitless injections into a 30-m length, 5 % phenylmethylsilicone fused silica capillary column (DB-5MS, Agilent Technologies, Palo Alto, CA, USA) with a 10-m length, integrated, deactivated guard column. Regarding the three quadrupoles in the Bruker Mass Spectrometer, we used one quadrupole as mass filter and ran the instrument in single ion monitoring mode, which helped to improve the detection and quantification of the PAHs especially for the alkylated isomers.

We ran three blanks (*n* = 3) prior to each sequence of samples and average values from those blanks were subtracted from the sample data later. Six-point internal calibration (ranging from 0.5 to 5 ng μL^−1^) curves were run prior to the GC–MS analyses of BB samples (R^2^ = 0.991–0.998). Each PAH concentration was calculated based on relative concentration of its deuterated PAH analog (or of a PAH with similar response factor). For quality assurance purposes, two different calibration levels were ran after each 10 samples. Replicate precision of the GC-MS method was ~10 % for all analyzed PAHs. The limit of detection (LOD) was obtained for the 16 EPA PAHs and it varied between 0.02 and 0.05 ng μL^−1^. The limit of quantification (LOQ) for PAHs was calculated by multiplying the LOD by 3.3 (U. S. Food and Drug Administration/Center for Biologics Evaluation and Research, 1995) and thereby the LOQ for analyzed PAHs was in the range of 0.066 and 0.165 ng μL^−1^.

## 3. Results and discussion

### 3.1. High gas-phase emission

**Fig. 1.**
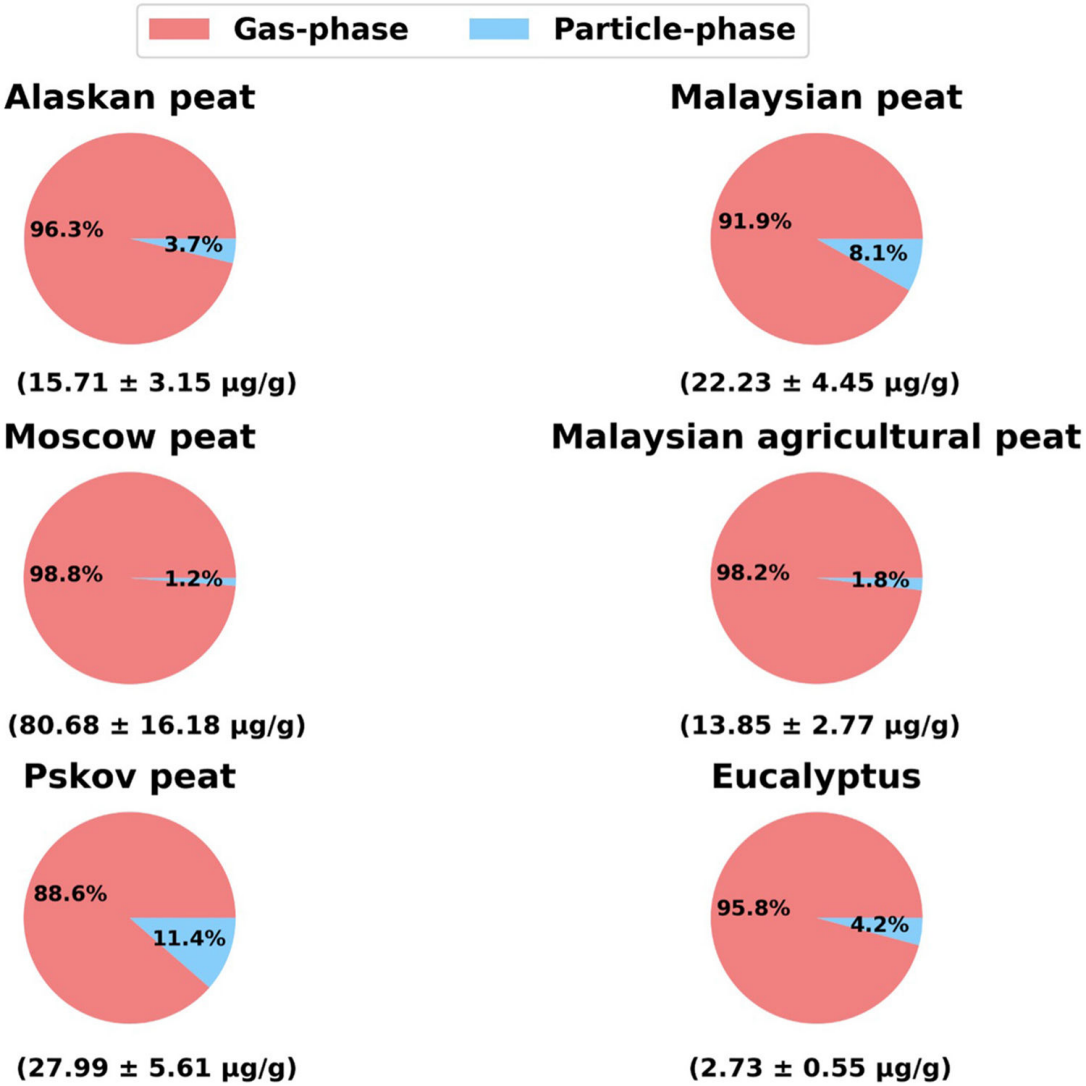
Fractional mass of PAHs in gas- and particle-phase emissions from combustion of six different fuels: Alaskan peat, Malaysian peat, Moscow peat, Malaysian agricultural peat, Pskov peat, and Eucalyptus. Values in brackets represent the total emission factors (EFs) for both gas- and particle-phase PAH emission (total 113 PAHs) in pg of PAHs emitted per g of fuel burnt (μg g^−1^). Standard deviations were calculated based on replicate burn experiments and PAH analysis for similar fuels from our previous combustion chamber study (Samburova et al., 2016).

The contribution of gas- (collected on XAD resins) and particle-phase (collected on TIGF filters) PAHs and their total concentration in combustion emissions from six fuels are presented in Fig. 1. The relative distribution of semi-volatile compounds in gas and particle phase depends on dilution of the smoke plume, primarily due to dilution driven evaporation and subsequent chemistry (Hodshire et al., 2019, 2021). Thus, the observed gas-particle partitioning reported here is specific for the experimental conditions employed in our study. To extrapolate our results to other dilution conditions, the Pankow gas-partitioning theory can be used (Pankow, 1994). From the partitioning theory it follows that the ratio of compound's gas concentration to its concentration in the particle phase is proportional to its saturation vapor concentration and inversely proportional to the total aerosol mass in which the substance can dissolve. Even though the saturation concentrations are not known for most of the compounds measured in our study, their gas particle partitioning can be extrapolated to other dilution ratios using this relationship. For example, if the aerosol was diluted such that its concentration was reduced by a factor of 10 relative to our experiments, the gas to particle ratio of each compound will increase by a factor of 10 relative to out measurements. In our measurements, the total aerosol concentrations were 19.6 mg m^−3^ for Eucalyptus and 29.7 mg m^−3^ for Malaysian peat.

Considering fresh biomass-burning emissions for all fuels, gas-phase PAHs are significantly more abundant (8–80 times) by mass than particle-phase PAHs. The fractional mass of gas-phase PAHs is between 89 and 99 % (Fig. 1), with the lower end of this range being consistent with that for emissions from the combustion of pine (89.6 %), oak (88.3 %), and eucalyptus (86.2 %), previously reported by Schauer et al. (2001). For our study, the fractional mass of gas-phase PAHs is on the higher end of the range reported by Schauer et al. (2001); this is most likely due to the extended number of analyzed PAHs (113), including mono/di/tri substituted naphthalenes, which mainly appear in the gas phase (Fig. S1, (Samburova et al., 2017)). Substituted naphthalenes are discussed in more detail in Section 3.3. The total emission factors (EFs) for the 113 analyzed PAHs are higher for smoldering combustion of peat samples (for example, Pskov peat total PAHs~28 μg g^−1^) compared to flaming combustion of Eucalyptus (total PAHs~2.7 μg g^−1^). As the burning conditions shift from smoldering to flaming combustion (Jenkins et al., 1996), the combustion efficiency increases with it approaching one for complete flaming combustion. Increasing combustion efficiency leads to a decrease in overall OC emissions including those of PAHs. Similar results were observed by Iinuma et al. (2007) in their laboratory analysis of 28 PAHs. Black et al. (2016) and Iinuma et al. (2007) reported the average value of total PAH EFs for combustion of both boreal and tropical peat land to be around 20 μg g^−1^.

### 3.2. Gas- vs. particle-phase emissions for different aromatic-ring PAHs

**Fig. 2.**
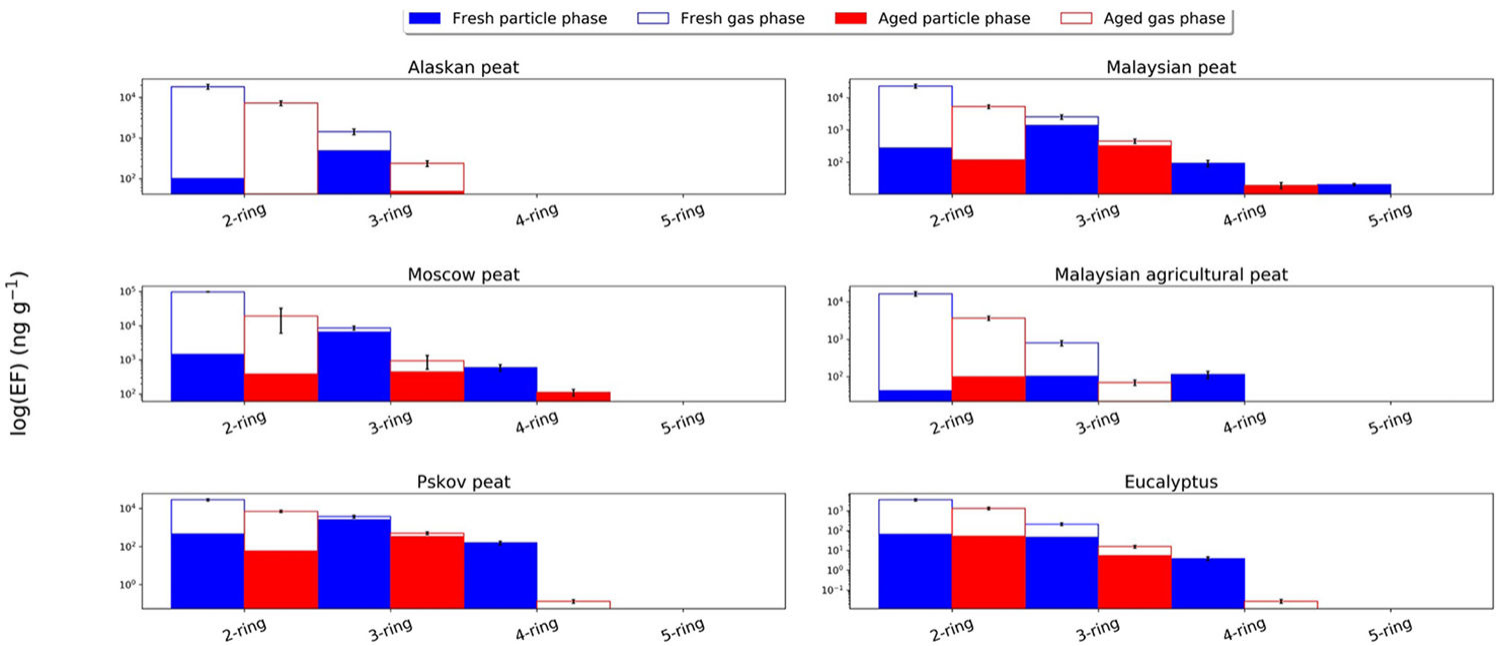
Fuel-based emission factors (EFs) of 2–5 ring PAHs for combustion of six fuels: Alaskan peat, Malaysian peat, Moscow peat, Malaysian agricultural peat, Pskov peat, and eucalyptus. EFs are presented separately for gas-(open bars) and particle-(solid bars) phase species and for fresh (blue) and OFR-aged (red) emissions, all on logarithmic scales. EF units: ng g^−1^; mass of PAH emission per fuel mass consumed. The error bars represent standard-deviation of EFs for analyzed PAHs that were calculated based on replicate analysis of EFs from similar fuels (with the same experimental conditions) from our previous campaign based on the data reported by Samburova et al. (2016).

Fig. 2 shows the EFs of 2- to 5-aromatic ring PAHs (6-aromatic and 7-aromatic ring PAHs were below detection limit in the current study) in both gas (open bars) and particle phase (solid bars) for both fresh and OFR-aged emissions from combustion of the six fuels. High abundances of 2- and 3-aromatic ring PAHs were observed for all fuels. As was shown in Fig. 1, gas-phase PAHs have higher EFs than particle-phase PAHs and thus the dominance of 2- and 3-ring PAHs was anticipated (Fig. 2). For example, for combustion of Alaskan peat, the EF for 2-ring gas-phase PAHs in fresh emission is 18.2 μg g^−1^, which is almost 180 times higher than that for particle-phase 2-ring PAHs (0.1 μg g^−1^).

In this section, all comparisons and subsequent conclusions are derived from the 2- and 3-ring PAHs identified by our analytical method and do not completely account for all the 2- and 3-ring PAHs that are likely to be found in BB emissions. For fresh combustion emissions of Moscow peat and Eucalyptus, EFs for 2-ring PAHs in the gas phase were ~ 67 and ~ 55 times higher, respectively than EFs for those in the particle phase. On the other hand, for 3-ring PAHs, the difference between fresh gas- and particle-phase EFs is not as significant: ~2 times for Alaskan peat and ~3.7 times for Eucalyptus combustion emissions. In case of Moscow peat, the fresh EF for the particle-phase 3-ring PAHs (6.50 μg g^−1^) was ~3 times higher than the EF for those in gas phase (2.09 μg g^−1^) (Table S1). As was expected, 4-ring PAHs were emitted predominantly in the particle phase with highest EF for Moscow peat 0.59 μg g^−1^ (Table S1). 5-ring particle-phase PAHs were only detected in Malaysian peat emission with an EF of 20 ng g^−1^.

To assess the fractional mass contribution of different aromatic ring PAHs, the EFs of particle-phase PAHs were normalized to total particle-phase PAH EFs for each fuel (Fig. S1). In case of fresh BB emissions, 3-ring PAHs were the most abundant for particle phase emissions from combustion of all fuels except for Malaysian agricultural peat, where 4-ring PAHs contributed the most to the particle-phase PAH mass emissions (~44 %). Irrespective of geographical origin of peat fuels (mid latitude or tropical), the fractional contribution of 3-ring PAHs for all peats ranged from 75 to 83 % and this is characteristic of smoldering combustion. Eucalyptus, our fuel representative of flaming combustion, has a dominant mass contribution from 2-ring PAHs (~57 %, Fig. S1) to particle phase emissions. Previous work on combustion of eucalyptus fuel (Schauer et al., 2001) also reported high abundance of low number aromatic ring PAHs, however, only 3-ring PAHs were measured and reported as dominant PAHs (>40 % of mass). Combustion of other flaming fuels, for example pine, demonstrated the largest PAH contribution to combustion emissions from 4-aromatic ring PAHs (>45 % of mass) (Fine et al., 2001). However, this study didn’t report 2-ring PAHs either. Our results show that in our study low molecular weight substituted PAHs were important contributors to the total PAH mass.

Other combustion emissions, such as coal combustion or heavy-duty diesel emissions, also have their characteristic signature in relative contribution of different number of aromatic ring PAHs towards the total PAH emissions. For example, coal combustion (Masclet et al., 1987) and heavy duty diesel engine PAH mass emissions are dominated by 4-ring PAHs (Rogge et al., 1993; Zielinska et al., 2004), while gasoline combustion emissions mostly contain 4-, 5-, and 6-ring PAHs (Lima et al., 2005; Zielinska et al., 2004). In our analysis of BB emissions, we have not observed any 5-, and 6-ring PAHs.

Due to the complex matrix of numerous organic compounds in BB emissions (Mazzoleni et al., 2007; Simoneit, 2002), understanding the chemical transformation of PAHs in an OFR is a very challenging task. Overall, our results on OFR oxidation of BB emissions show that both gas- and particle-phase PAH emissions were reduced after OFR oxidation (Fig. 2). For example, after OFR oxidation of Malaysian peat combustion emissions, the EFs of gas-phase 2-ring PAHs decreased ~4.5 times, while for 3-ring particle-phase PAHs the decrease was almost 8 times. (Fig. 2).

Comparison of fractional contributions between fresh and OFR-aged particle emissions of different size PAHs can provide insight into potential processes occurring during OFR oxidation. Our results show that the fractional contribution of 3-ring particle-phase PAHs generally decreased during OFR-aging (with the exception of emissions from Pskov peat), whereas 2-ring PAHs' contribution to total PAHs increased (Fig. S1). For example, in case of Alaskan peat combustion, the contribution of 3-ring particle-phase PAHs to the total PAH mass decreased from 83 % to 54 %, while the contribution of 2-ring PAHs increased from 17 % to 46 %. Similarly, for eucalyptus combustion particle-phase emissions, the contribution of 3-ring PAHs decreased from 40 % to 9 %, while it increased from 57 % to 90 % for 2-ring PAHs. A more detailed characterization of PAH chemistry after OFR aging is still needed for further investigation and modeling of their fate during atmospheric transport and oxidations.

### 3.3. Substituted naphthalenes in gas phase

**Fig. 3.**
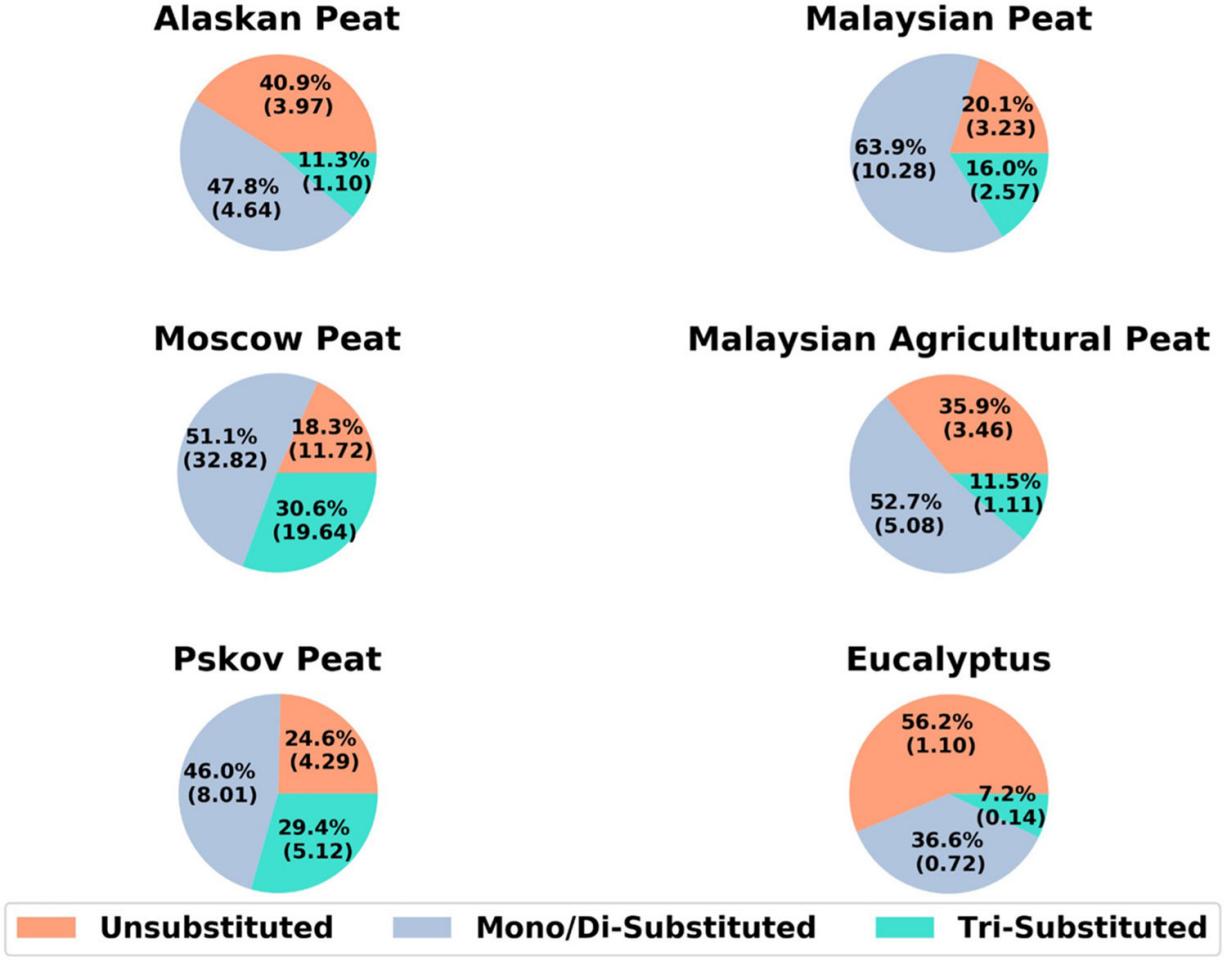
Fractional mass of unsubstituted and substituted naphthalenes in gas-phase emissions from combustion of six fuels; values in brackets represent the total emission factors (EFs) of analyzed naphthalenes in μg g^−1^ of fuel (μg g^−1^).

Fig. 3 shows the fractional mass distribution and EFs of unsubstituted and substituted naphthalenes (e.g., 1,3-dimethylnaphthalene) in the gas-phase emissions from combustion of the six fuels. For all fuels, except eucalyptus, the unsubstituted PAHs constitute between 18.3 and 40.9 % (Table S2) of the total analyzed gas-phase naphthalenes' mass. For eucalypus, which was the only fuel representative of flaming combustion, unsubstituted naphthalenes contributed >50 % of the total analyzed gas-phase naphthalenes' mass.

The high abundances of substituted naphthalenes (di- and tri-methyl naphthalenes) in peat combustion aerosols, relative to non-substituted ones, can potentially be explained by lower combustion temperature and/or evaporation of low-volatility, petrogenic PAHs from peat fuel. Peats are formed in the rudimentary stages of the overall diagenetic process of coal formation (Bustin, 1998). PAHs are found to be present in coal even without any sort of combustion (Achten and Hofmann, 2009; Liu et al., 2008). The presence of substituted PAHs is not limited to coal. In a recent study, involving ambient monitoring of sites adjacent to Canadian oil sands, high abundance of alkyl-substituted PAHs was found and the origin of those alkyl-substituted PAHs was assumed to be petrogenic instead of conventional combustion sources (Hamer et al., 2018). Based on the evidence from coal and oil sands, we suspect that these alkyl-substituted naphthalenes may also be present in peats and evaporate before ignition and hence present in combustion emissions mostly in the gas phase. However, this study did not attempt to measure their in-situ formation during combustion versus what was present in the fuel. Considering the potential air quality impacts from increased frequency of both arctic and tropical peat fires (Hu et al., 2018) and the potential toxicity of alkyl-substituted PAHs (Andersson and Achten, 2015), detailed characterization of BB PAH chemistry, especially for substituted PAHs in both gas- and particle-phase PAHs, is very important.

### 3.4. Fate of individual compounds during OFR oxidation

Here, we discuss the fate of individual organic compounds during OFR oxidation. We will begin our discussion with unsubstituted PAH emissions and their transformations during OFR-aging by reporting the fractional change in total emissions (both gas and particle phase). However, for more explicit representation, gas- and particle-phase EFs are plotted separately with open and closed bars, respectively. A similar approach was taken for describing the fate of both substituted naphthalenes and substituted phenanthrenes as they were more abundant in the gas particle mixture than unsubstituted compounds in our analysis.

We have discussed earlier in the manuscript that 2- and 3-ring PAHs are found in high abundances in BB emissions. The abundances of 2- and 3-ring PAHs tends to decrease after OFR-aging (Fig. 2). When we zoomed into individual PAHs and most importantly Environmental Protection Agency (EPA) priority PAHs (USEPA, 1999), we did observe the same trend. For example, naphthalene, which is predominantly found in gas phase, decreased between 60 and 75 % after OFR oxidation for all analyzed fuels (Fig. 4a, Table S3). After OFR oxidation, we have not detected 1,4-napthaquinone (Fig. S4) which is potentially considered as naphthalene oxidation product that can form via hydrogen abstraction mechanism (electrophilic aromatic substitution) and without ring opening (Huang et al., 2019). The absence of aromatic substitution product formation during our analysis indicates that multiple generation of oxidation within the OFR environment leads to ring opening.

Retene, pyrene, and fluoranthene were primarily found in particle phase and these 4-ring compounds decreased from 57 % or even 100 % during OFR aging (Fig. 4a, Table S3). These 4-ring PAHs can undergo heterogenous oxidation with OH radical and/or ozone, in presence or absence of NO_x_, and potentially form oxo- and nitro-PAHs (Cochran et al., 2016a). Analysis of nitro-PAHs was beyond the scope of this study and from our current analysis, we are unable to identify any oxo-PAHs that can be directly related to those 4-ring parent PAHs (Fig. S4). Phenanthrene and anthracene and their substituted analogs can exist in both gas and particle phase (Atkinson and Arey, 1994). Both phenanthrene and anthracene decreased after OFR oxidation (Fig. 4a). However, the extent of decrease for anthracene is higher (almost 100 %) than that for phenanthrene (65–90 % decrease) as expected from relative rate constant values (Nicovich et al., 1981). A similar trend is observed for substituted phenanthrene and anthracene compounds (Fig. S5). We believe that direct reactions between gas-phase oxidants and particle-phase organic molecules are highly unlikely inside the OFR, because heterogeneous reactions with OH radicals are significantly (orders of magnitudes) slower than gas-phase reactions (Kroll et al., 2015). We expect that these particle phenanthrenes, due to their semi-volatile nature, steadily evaporated as they reacted in the gas phase to form oxo/nitro-PAHs and other oxidized products (Kwok et al., 1994; Zeng et al., 2020; Zhao et al., 2016). However, we also identified a potential artifact of our oxidation setup that in absence of an ozone scrubber in front of the tandem filter-XAD collection system, heterogeneous oxidation with ozone can take place on the filter surface (Sengupta et al., 2020) and thus can potentially impact fate of PAHs.

We have highlighted the importance of substituted naphthalenes earlier as we demonstrated that 2-methylnapthalene, 1-methylnapthalene, and 1- and 2-ethylnapthalenes are the most abundant compounds (15–20 % of total mass of analyzed gas-phase PAHs) for analyzed BB emissions (Fig. 3). After the OFR aging, 2-methylnapthalene was reduced by 75 % for Alaskan peat emissions and by 82 % for Moscow peat emissions (Fig. 4b). A similar extent of EF reduction was observed for 1-methylnapthalene (78 % for Eucalyptus, 80 % for Malaysian peat) and for 1 + 2-ethylnapthalenes (74 % for Pskov peat, 94 % for Malaysian agricultural peat). Other di-substituted naphthalenes demonstrated similar trends in reduction during the OFR oxidation. For example, 1,3 + 1,6 + 1,7-dimethylnaphthalene EF was reduced by 86 % for Alaskan peat. During oxidation processes, PAHs can undergo decomposition via electrophilic aromatic substitution in the presence of OH radicals (Shrivastava et al., 2017; Zhou et al., 2019), which is considered to be the most predominant and active oxidant generated in OFR (Bhattarai et al., 2018; Palm et al., 2016). The oxidation process can cause the formation of oxo-PAHs such as hydroxy-biphenyl, 2-formylcinnamaldehydes, substituted-1,4-napthaquinone, while going through some transient intermediate products like naphthols (Atkinson et al., 1987; Bunce et al., 1997; Sasaki et al., 1998). Oxidized products from naphthalene and substituted naphthalenes tend to partition to the particle phase and contribute towards SOA yield (Chan et al., 2009) and hence it will be really important to conduct future OFR (or any other flow reactor based) studies involving PAH mixtures and covering multiple oxidation regimes that can give us more insight into the fate and transformation processes of PAHs in the atmosphere.

### 3.5. TEFs of 88 analyzed PAH

Risk assessment and potential toxicity evaluation for a mixture of PAH compounds is usually performed by converting the contributions from individual PAHs to benzo-a-pyrene equivalent (BaP_eq_) dose (Larsen and Larsen, 1998). In most of the previous toxicity evaluation studies, the BaP_eq_ TEFs were calculated for the particle-bound 16 US EPA priority PAHs (Wu et al., 2022) and very few studies (Ramírez et al., 2011; Samburova et al., 2016) have considered the contributions of gas-phase PAHs towards overall toxicity of BB smoke. Here, we calculated the PAH toxicity of 88 BB PAHs for both gas and particle phases following Samburova et al. (2017). TEF assignments and the change in total PAH toxicity of the BB samples was assessed after OFR oxidation. The total PAH toxicity calculated (Eq. 1 and Table S4 in the supplement section) with 88 analyzed PAHs demonstrated a decrease (Fig. S6) for BB emissions from all six fuels after OFR-aging. On the other hand, it has been reported that atmospheric aging can increase both oxidative potential and cytotoxicity of BB smoke (Fan et al., 2020; Wong et al., 2019). Therefore, the increase in the toxicity of the aged BB emissions may be explained by the formation of other toxic products including PAH derivatives such as oxo-PAHs (Cochran et al., 2016b; Hayakawa et al., 2017; Visentin et al., 2016). In the current study, we emphasize that it is focused only on PAH toxicity instead of total toxicity (i.e., toxicity including PAH oxidation products).

**Fig. 5.**
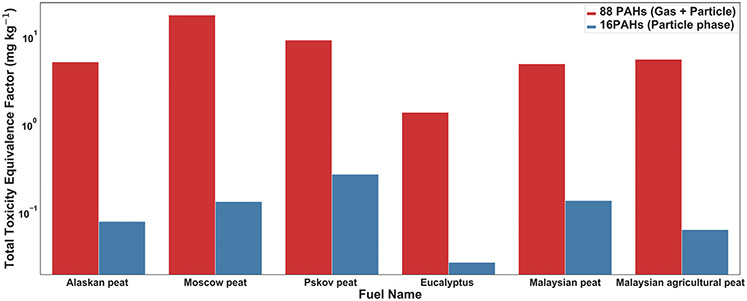
Toxicity Equivalence Factors (TEFs) calculated for fresh BB emissions from six important biomass fuels.

Following Samburova et al. (2017), we compared the total BaP_eq_ TEFs of 88 PAHs (sum of gas- and particle-phase PAHs) with those for 16 particle-phase EPA PAHs (USEPA, 1999). Our results indicate that the PAH toxicity of BB samples is underestimated by 10–100 times if only the BaP_eq_ for the 16 US EPA priority PAHs in the particle phase are included (Fig. 5).

Overall, our laboratory BB burning experiments showed that the total BaP_eq_ TEF (sum of gas- and particle-phase BaP_eq_ TEFs) for each fuel varied from 2 mg kg^−1^ to 13 mg kg^−1^. In comparison, previous studies reported from 30 mg kg^−1^ to 105 mg kg^−1^ of total BaP_eq_ TEFs for household coal and other BB fuels, which was also controlled by combustion efficiency (Wu et al., 2022). Coal and household emissions can potentially contain a higher fraction of 4/5-ring PAHs that have higher individual toxic potencies than 2/3-ring PAHs (mostly found in BB emissions by this analysis), and hence the relatively lower values of total BaP_eq_ TEFs from peat and Eucalyptus burning are reconcilable. For improved health risk assessments and policy decisions, we strongly recommend to include the TEFs measured for both gas- and particle-phase PAHs beyond the 16 US EPA priority PAHs and to report toxicity weighted emission factors from multiple combustion sources.

## 4. Conclusions

The toxicity of PAHs drives the attention of the scientific community towards extensive chemical characterization and understanding the potential fate of this class of compounds. In our study, we have highlighted that the traditional matrix of characterization (16 EPA PAHs in particle phase) could underestimate the total PAH toxicity of BB emissions by 10–100 times depending on fuels and burning conditions. With our extensive characterization work (with 88 PAHs for which TEF was calculated), we were able to understand “what is missing” and “what needs to be characterized further”. PAHs in BB and especially in smoldering emissions from combustion of peat fuel are primarily found in gas phase and hence low molecular weight 2/3-ring PAHs are prevalent over 4/5-ring PAHs which are more abundant in diesel particle emissions. Quantitative analysis of individual PAHs showed that substituted naphthalene and phenanthrenes are key contributors to the PAH toxicity which is reflected in overall TEF values (10–100 times higher than that estimated with only 16 EPA priority PAHs) for analyzed fuels. Substituted naphthalene and phenanthrenes have lower individual toxic potencies (BaP_eq_ TEFs) than 4/5-ring PAHs. However, their high abundance in gas phase (which was not traditionally characterized in previous studies) makes them the major contributors to the overall TEF values. We have observed decrease in PAH concentrations after the OFR aging that agrees well with the relative rate constant values provided by previous lab studies (Nayebzadeh and Vahedpour, 2017; Nicovich et al., 1981). Based on the relative abundance of oxo-PAHs in fresh and aged BB emissions, we observed a decrease in oxo-PAHs, which are potentially more toxic than their parent analogs (Geier et al., 2018). However, from our study, we were unable to determine whether the overall toxicity of combustion emissions increased or decreased after OFR-aging. So, we recommend future laboratory-based comprehensive studies including multiple gas and particle phase PAHs of combustion origin that would potentially combine detailed chemical characterization and toxicity potential measurements to understand the fate of PAHs (substitution vs. ring opening) and hence their contribution to potential toxicity after aging/atmospheric oxidation.

## Supplementary Material

Supplementary material

## Figures and Tables

**Fig. 4a.**
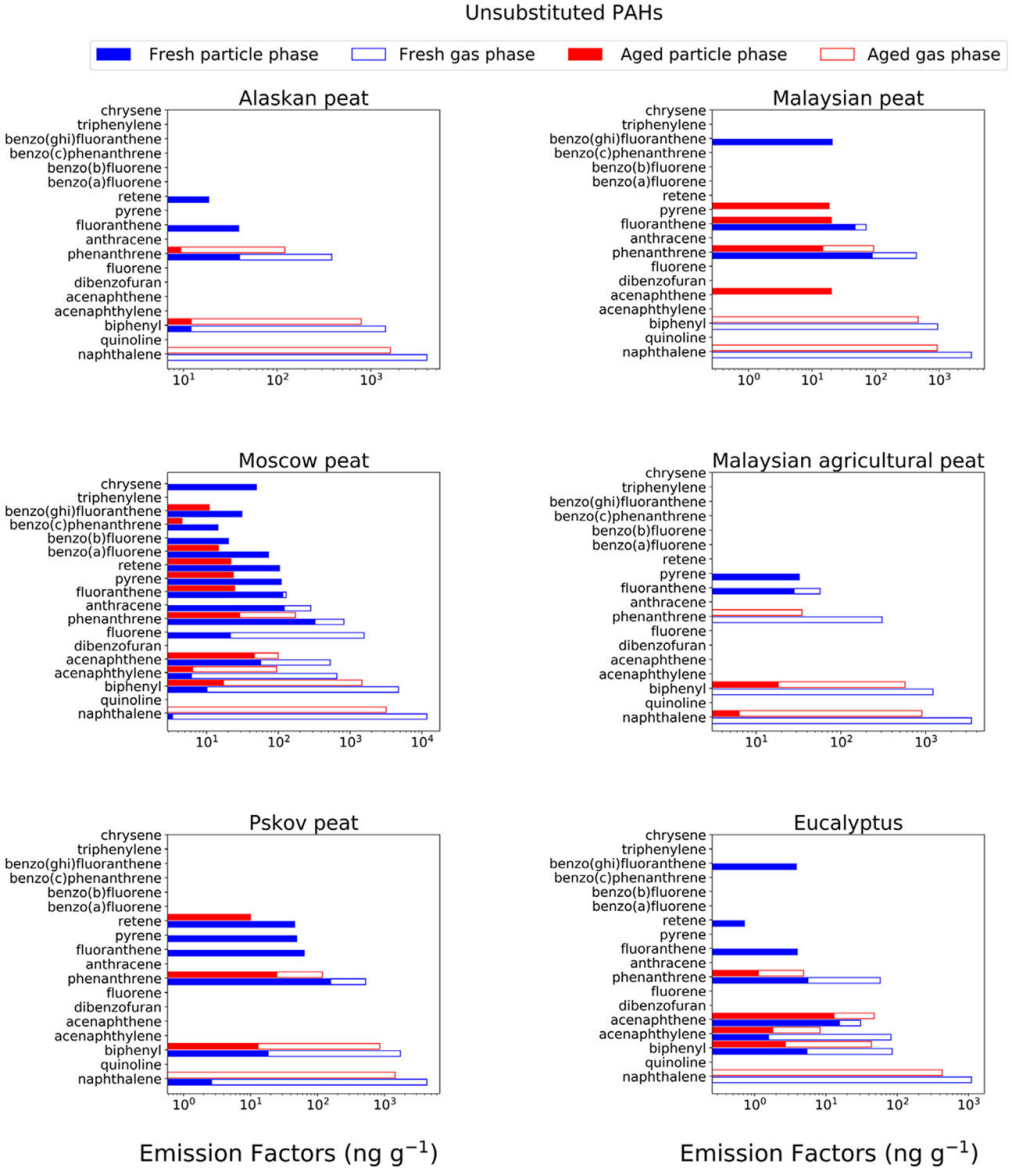
EFs (on log scales) of unsubstituted PAHs for combustion of six analyzed fuels are presented separately for gas-(open bars) and particle-(solid bars) phase species and for fresh (blue) and OFR-aged (red) emissions. Standard deviations of the EFs were calculated based on replicated burns performed by Samburova et al. (2016) and ranged between 46 % and 48 % for mono and disubstituted naphthalene (not shown in figure).

**Fig. 4b.**
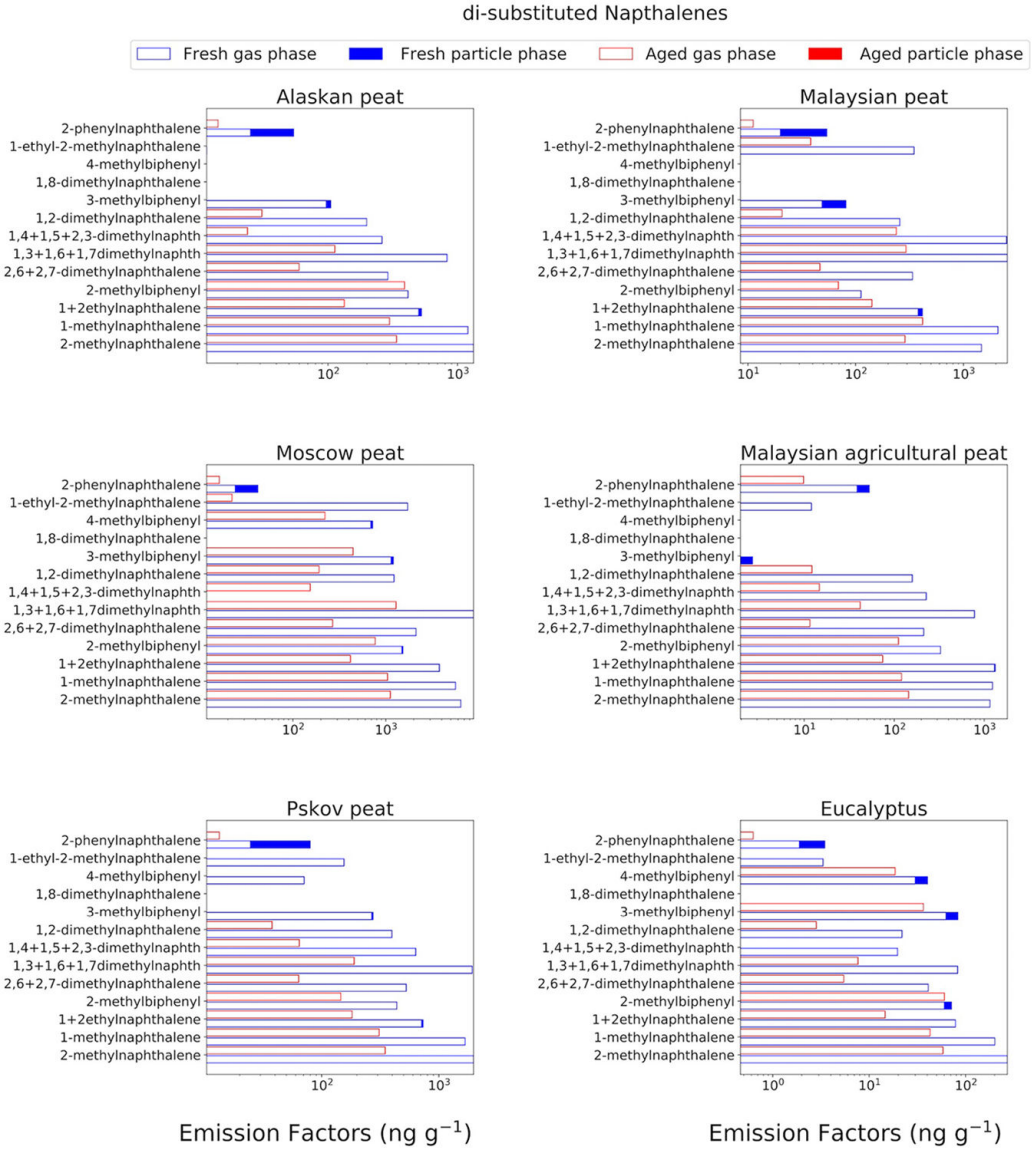
EFs (on log scales) of mono/di-substituted naphthalenes BB emissions for combustion of six different fuels presented separately for gas-(open bars) and particle-(solid bars) phase species and for fresh (blue) and OFR-aged (red) emissions. Standard deviations of the EFs were calculated based on replicated burns performed by Samburova et al. (2016) and ranged between 46 % and 48 % for mono and disubstituted naphthalenes (not shown in figure).

## Data Availability

Data will be made available on request.

## HIGHLIGHTS

- Emissions of biomass burning PAHs are important because of their potential toxicity
- Emissions of 113 PAHs and derivatives from combustion of six fuels were quantified
- We used Gas Chromatography-Mass Spectrometry for analysis of PAHs and PAH derivatives
- Analyzing only particle phase 16 EPA priority PAHs may greatly underestimate toxicity
- Characterization of gas phase of PAHs is of the essence for PAH toxicity evaluation

## References

[R1] Achten C, Hofmann T, 2009. Native polycyclic aromatic hydrocarbons (PAH) in coals - a hardly recognized source of environmental contamination. Sci. Total Environ 407, 2461–2473. 10.1016/j.scitotenv.2008.12.008. 19195680

[R2] Akagi SK, Yokelson RJ, Wiedinmyer C, Alvarado MJ, Reid JS, Karl T, Crounse JD, Wennberg PO, 2011. Emission factors for open and domestic biomass burning for use in atmospheric models. Atmos. Chem. Phys 11, 4039–4072. 10.5194/acp-11-4039-2011.

[R3] Ali MU, Siyi L, Yousaf B, Abbas Q, Hameed R, Zheng C, Kuang X, Wong MH, 2021. Emission sources and full spectrum of health impacts of black carbon associated polycyclic aromatic hydrocarbons (PAHs) in urban environment: a review. Crit. Rev. Environ. Sci. Technol 51, 857–896. 10.1080/10643389.2020.1738854.

[R4] Andersson JT, Achten C, 2015. Time to say goodbye to the 16 EPA PAHs? Toward an up-to-date use of PACs for environmental purposes. Polycycl. Aromat. Compd 35, 330–354. 10.1080/10406638.2014.991042. 26823645 PMC4714241

[R5] Andreae MO, 2019. Emission of trace gases and aerosols from biomass burning – an updated assessment. Atmos. Chem. Phys 19, 8523–8546. 10.5194/acp-19-8523-2019.

[R6] Andreae MO, Merlet P, 2001. Emission of trace gases and aerosols from biomass burning. Glob. Biogeochem. Cycles 15, 955–966. 10.1029/2000GB001382.

[R7] Apte JS, Marshall JD, Cohen AJ, Brauer M, 2015. Addressing global mortality from ambient PM2.5. Environ. Sci. Technol 49, 8057–8066. 10.1021/acs.est.5b01236. 26077815

[R8] Atkinson R, Arey J, 1994. Atmospheric chemistry of gas-phase polycyclic aromatic hydrocarbons: formation of atmospheric mutagens. Environ. Health Perspect 102, 117–126. 10.2307/3431940. PMC1566940 7821285

[R9] Atkinson R, Arey J, Zielinska B, Aschmann SM, 1987. Kinetics and products of the gas-phase reactions of OH radicals and N2O5 with naphthalene and biphenyl. Environ. Sci. Technol 21, 1014–1022. 10.1021/es50001a017. 19995002

[R10] Bandowe BAM, Meusel H, 2017. Nitrated polycyclic aromatic hydrocarbons (nitro-PAHs) in the environment – a review. Sci. Total Environ 581–582, 237–257. 10.1016/j.scitotenv.2016.12.115. 28069306

[R11] Bhattarai C, Samburova V, Sengupta D, Iaukea-Lum M, Watts AC, Moosmüller H, Khlystov AY, 2018. Physical and chemical characterization of aerosol in fresh and aged emissions from open combustion of biomass fuels. Aerosol Sci. Technol 52, 1266–1282. 10.1080/02786826.2018.1498585.

[R12] Black RR, Aurell J, Holder A, George IJ, Gullett BK, Hays MD, … Tabor D, 2016. Characterization of gas and particle emissions from laboratory burns of peat. Atmos. Environ 132, 49–57.

[R13] Bunce NJ, Liu L, Zhu J, Lane DA, 1997. Reaction of naphthalene and its derivatives with hydroxyl radicals in the gas phase. Environ. Sci. Technol 31, 2252–2259. 10.1021/es960813g.

[R14] Bustin RM, 1998. Coal: Origin and Diagenesis BT - Geochemistry. Springer Netherlands, Dordrecht, pp. 90–92 10.1007/1-4020-4496-8_58.

[R15] Campos I, Abrantes N, Pereira P, Micaelo AC, Vale C, Keizer JJ, 2019. Forest fires as potential triggers for production and mobilization of polycyclic aromatic hydrocarbons to the terrestrial ecosystem. LandDegrad. Dev 30, 2360–2370. 10.1002/ldr.3427.

[R16] Cançado JED, Saldiva PHN, Pereira LAA, Lara LBLS, Artaxo P, Martinelli LA, Arbex MA, Zanobetti A, Braga ALF, 2006. The impact of sugar cane-burning emissions on the respiratory system of children and the elderly. Environ. Health Perspect 114, 725–729. 10.1289/ehp.8485. 16675427 PMC1459926

[R17] Chan AWH, Kautzman KE, Chhabra PS, Surratt JD, Chan MN, Crounse JD, Kürten A, Wennberg PO, Flagan RC, Seinfeld JH, 2009. Secondary organic aerosol formation from photooxidation of naphthalene and alkylnaphthalenes: implications for oxidation of intermediate volatility organic compounds (IVOCs). Atmos. Chem. Phys 9, 3049–3060. 10.5194/acp-9-3049-2009.

[R18] Chen J, Li C, Ristovski Z, Milic A, Gu Y, Islam MS, Wang S, Hao J, Zhang H, He C, Guo H, Fu H, Miljevic B, Morawska L, Thai P, Lam YF, Pereira G, Ding A, Huang X, Dumka UC, 2017. A review of biomass burning: emissions and impacts on air quality, health and climate in China. Sci. Total Environ 579, 1000–1034. 10.1016/j.scitotenv.2016.11.025. 27908624

[R19] Chen H, Chow AT, Li XW, Ni HG, Dahlgren RA, Zeng H, Wang JJ, 2018. Wildfire burn intensity affects the quantity and speciation of polycyclic aromatic hydrocarbons in soils. ACS Earth SpaceChem. 2, 1262–1270. 10.1021/acsearthspacechem.8b00101.

[R20] ChooChuay C, Pongpiachan S, Tipmanee D, Deelaman W, Iadtem N, Suttinun O, Wang Q, Xing L, Li G, Han Y, Hashmi MZ, Palakun J, Poshyachinda S, Aukkaravittayapun S, Surapipith V, Cao J, 2022. Effects of agricultural waste burning on PM2.5-bound polycyclic aromatic hydrocarbons, carbonaceous compositions, and water-soluble ionic species in the ambient air of Chiang-Mai, Thailand. Polycycl. Aromat. Compd 42, 749–770. 10.1080/10406638.2020.1750436.

[R21] Cochran RE, Jeong H, Haddadi S, Fisseha Derseh R, Gowan A, Beránek J, Kubátová A, 2016a. Identification of products formed during the heterogeneous nitration and ozonation of polycyclic aromatic hydrocarbons. Atmos. Environ 128, 92–103. 10.1016/j.atmosenv.2015.12.036.

[R22] Cochran RE, Smoliakova IP, Kubátová A, 2016b. Detection of nitrated and oxygenated polycyclic aromatic hydrocarbons using atmospheric pressure chemical ionization high resolution mass spectrometry. Int. J. Mass Spectrom 397–398, 6–17. 10.1016/j.ijms.2016.01.001.

[R23] Daellenbach KR, Uzu G, Jiang J, Cassagnes LE, Leni Z, Vlachou A, Stefenelli G, Canonaco F, Weber S, Segers A, Kuenen JJP, Schaap M, Favez O, Albinet A, Aksoyoglu S, Dommen J, Baltensperger U, Geiser M, El Haddad I, Jaffrezo JL, Prévôt ASH, 2020. Sources of particulate-matter air pollution and its oxidative potential in Europe. Nature 587, 414–419. 10.1038/s41586-020-2902-8. 33208962

[R24] Dhammapala R, Claiborn C, Jimenez J, Corkill J, Gullett B, Simpson C, Paulsen M, 2007. Emission factors of PAHs, methoxyphenols, levoglucosan, elemental carbon and organic carbon from simulated wheat and Kentucky bluegrass stubble burns. Atmos. Environ 41, 2660–2669. 10.1016/j.atmosenv.2006.11.023.

[R25] Dong TTT, Stock WD, Callan AC, Strandberg B, Hinwood AL, 2020. Emission factors and composition of PM2.5 from laboratory combustion of five Western Australian vegetation types. Sci. Total Environ 703, 134796. 10.1016/j.scitotenv.2019.134796. 31731149

[R26] Du W, Wang J, Zhuo S, Zhong Q, Wang W, Chen Y, Wang Z, Mao K, Huang Y, Shen G, Tao S, 2021. Emissions of particulate PAHs from solid fuel combustion in indoor cookstoves. Sci. Total Environ 771, 145411. 10.1016/j.scitotenv.2021.145411. 33524679

[R27] Faccinetto A, Desgroux P, Ziskind M, Therssen E, Focsa C, 2011. High-sensitivity detection of polycyclic aromatic hydrocarbons adsorbed onto soot particles using laser desorption/laser ionization/time-of-flight mass spectrometry: an approach to studying the soot inception process in low-pressure flames. Combust. Flame 158, 227–239. 10.1016/j.combustflame.2010.08.012.

[R28] Fan X, Cao T, Yu X, Wang Y, Xiao X, Li F, Xie Y, Ji W, Song J, Peng P, 2020. The evolutionary behavior of chromophoric brown carbon during ozone aging of fine particles from biomass burning. Atmos. Chem. Phys 20, 4593–4605. 10.5194/acp-20-4593-2020.

[R29] Fine PM, Cass GR, Simoneit BRT, 2001. Chemical characterization of fine particle emissions from fireplace combustion of woods grown in the northeastern United States. Environ. Sci. Technol 35, 2665–2675. 10.1021/es001466k. 11452590

[R30] Fortenberry CF, Walker MJ, Zhang Y, Mitroo D, Brune WH, Williams BJ, 2018. Bulk and molecular-level characterization of laboratory-aged biomass burning organic aerosol from oak leaf and heartwood fuels. Atmos. Chem. Phys 18, 2199–2224. 10.5194/acp-18-2199-2018.

[R31] Friedman CL, Selin NE, 2012. Long-range atmospheric transport ofpolycyclic aromatic hydrocarbons: a global 3-D model analysis including evaluation of arctic sources. Environ. Sci. Technol 46, 9501–9510. 10.1021/es301904d. 22856669

[R32] Friedman CL, Pierce JR, Selin NE, 2014. Assessing the influence of secondary organic versus primary carbonaceous aerosols on long-range atmospheric polycyclic aromatic hydrocarbon transport. Environ. Sci. Technol 48, 3293–3302. 10.1021/es405219r. 24564497

[R33] Geier MC, Chlebowski AC, Truong L, Massey Simonich SL, Anderson KA, Tanguay RL, 2018. Comparative developmental toxicity of a comprehensive suite of polycyclic aromatic hydrocarbons. Arch. Toxicol 92, 571–586. 10.1007/s00204-017-2068-9. 29094189 PMC5820187

[R34] Geng C, Chen J, Yang X, Ren L, Yin B, Liu X, Bai Z, 2014. Emission factors of polycyclic aromatic hydrocarbons from domestic coal combustion in China. J. Environ. Sci. (China) 26, 160–166. 10.1016/S1001-0742(13)60393-9. 24649702

[R35] Harner T, Rauert C, Muir D, Schuster JK, Hsu YM, Zhang L, Marson G, Watson JG, Ahad J, Cho S, Jariyasopit N, Kirk J, Korosi J, Landis MS, Martin JW, Zhang Y, Fernie K, Wentworth GR, Wnorowski A, Dabek E, Charland JP, Pauli B, Wania F, Galarneau E, Cheng I, Makar P, Whaley C, Chow JC, Wang X, 2018. Air synthesis review: polycyclic aromatic compounds in the oil sands region. Environ. Rev 26, 430–468. 10.1139/er-2018-0039.

[R36] Harris RMB, Remenyi TA, Williamson GJ, Bindoff NL, Bowman DMJS, 2016. Climate–vegetation–fire interactions and feedbacks: trivial detail or major barrier to projecting the future of the Earth system? Wiley Interdiscip.Rev. Clim. Chang 7, 910–931. 10.1002/wcc.428.

[R37] Hayakawa K, Tang N, Toriba A, 2017. Recent analytical methods for atmospheric polycyclic aromatic hydrocarbons and their derivatives. Biomed. Chromatogr 31, 1–10. 10.1002/bmc.3862. 27723111

[R38] He J, Zielinska B, Balasubramanian R, 2010. Composition of semi-volatile organic compounds in the urban atmosphere of Singapore: influence of biomass burning. Atmos. Chem. Phys 10, 11401–11413. 10.5194/acp-10-11401-2010.

[R39] Hodshire AL, Akherati A, Alvarado MJ, Brown-Steiner B, Jathar SH, Jimenez JL, Kreidenweis SM, Lonsdale CR, Onasch TB, Ortega AM, Pierce JR, 2019. Aging effects on biomass burning aerosol mass and composition: a critical review of field and laboratory studies. Environ. Sci. Technol 53, 10007–10022. 10.1021/acs.est.9b02588. 31365241

[R40] Hodshire AL, Ramnarine E, Akherati A, Alvarado ML, Farmer DK, Jathar SH, Kreidenweis SM, Lonsdale CR, Onasch TB, Springston SR, Wang J, Wang Y, Kleinman LI, Sedlacek AJ III, Pierce JR, 2021. Dilution impacts on smoke aging: evidence in Biomass Burning Observation Project (BBOP) data. Atmos. Chem. Phys 21, 6839–6855. 10.5194/acp-21-6839-2021.

[R41] Holden ZA, Swanson A, Luce CH, Jolly WM, Maneta M, Oyler JW, Warren DA, Parsons R, Affleck D, 2018. Decreasing fire season precipitation increased recent western US forest wildfire activity. Proc. Natl. Acad. Sci. U. S. A 115, E8349–E8357. 10.1073/pnas.1802316115. 30126983 PMC6130364

[R42] Hu Y, Fernandez-Anez N, Smith TEL, Rein G, 2018. Review of emissions from smouldering peat fires and their contribution to regional haze episodes. Int. J. Wildl. Fire 27, 293–312. 10.1071/WF17084.

[R43] Huang G, Liu Ying, Shao M, Li Y, Chen Q, Zheng Y, Wu Z, Liu Yuechen, Wu Y, Hu M, Li X, Lu S, Wang C, Liu J, Zheng M, Zhu T, 2019. Potentially important contribution of gas-phase oxidation of naphthalene and methylnaphthalene to secondary organic aerosol during haze events in Beijing. Environ. Sci. Technol 53, 1235–1244. 10.1021/acs.est.8b04523. 30625271

[R44] Hurteau MD, Westerling AL, Wiedinmyer C, Bryant BP, 2014. Projected effects of climate and development on California wildfire emissions through 2100. Environ. Sci. Technol 48, 2298–2304. 10.1021/es4050133. 24443984

[R45] Iinuma Y, Brüggemann E, Gnauk T, Müller K, Andreae MO, Helas G, Parmar R, Herrmann H, 2007. Source characterization of biomass burning particles: the combustion of selected European conifers, African hardwood, savanna grass, and German and Indonesian peat. J. Geophys. Res. Atmos 112. 10.1029/2006JD007120.

[R46] Jaffe D, Bertschi I, Jaeglé L, Novelli P, Reid JS, Tanimoto H, Vingarzan R, Westphal DL, 2004. Long-range transport of Siberian biomass burning emissions and impact on surface ozone in western North America. Geophys. Res. Lett 31, 6–9. 10.1029/2004GL020093.

[R47] Jenkins BM, Jones AD, Turn SQ, Williams RB, 1996. Emission factors for polycyclic aromatic hydrocarbons from biomass burning. Environ. Sci. Technol 30, 2462–2469. 10.1021/es950699m.

[R48] Johnston FH, Henderson SB, Chen Y, Randerson JT, Marlier M, DeFries RS, Kinney P, Bowman DMJS, Brauer M, 2012. Estimated global mortality attributable to smoke from landscape fires. Environ. Health Perspect 120, 695–701. 10.1289/ehp.1104422. 22456494 PMC3346787

[R49] Jolly WM, Cochrane MA, Freeborn PH, Holden ZA, Brown TJ, Williamson GJ, Bowman DMJS, 2015. Climate-induced variations in global wildfire danger from 1979 to 2013. Nat. Commun 6, 1–11. 10.1038/ncomms8537. PMC4803474 26172867

[R50] Kim Oanh NT, Nghiem LH, Phyu YL, 2002. Emission of polycyclic aromatic hydrocarbons, toxicity, and mutagenicity from domestic cooking using sawdust briquettes, wood, and kerosene. Environ. Sci. Technol 36, 833–839. 10.1021/es011060n. 11918004

[R51] Kim IS, Lee JY, Kim YP, 2013. Impact of polycyclic aromatic hydrocarbon (PAH) emissions from North Korea to the air quality in the Seoul Metropolitan Area, South Korea. Atmos. Environ 70, 159–165. 10.1016/j.atmosenv.2012.12.040.

[R52] Kojima Y, Inazu K, Hisamatsu Y, Okochi H, Baba T, Nagoya T, 2010. Influence of secondary formation on atmospheric occurrences of oxygenated polycyclic aromatic hydrocarbons in airborne particles. Atmos. Environ 44, 2873–2880. 10.1016/j.atmosenv.2010.04.048.

[R53] Kroll JH, Lim CY, Kessler SH, Wilson KR, 2015. Heterogeneous oxidation of atmospheric organic aerosol: kinetics of changes to the amount and oxidation state of particle-phase organic carbon. J. Phys. Chem. A 119, 10767–10783. 10.1021/acs.jpca.5b06946. 26381466

[R54] Kwok ESC, Harger WP, Arey J, Atkinson R, 1994. Reactions of gas-phase phenanthrene under simulated atmospheric conditions. Environ. Sci. Technol 28, 521–527. 10.1021/es00052a027. 22165890

[R55] Larsen JC, Larsen PB, 1998. Chemical carcinogens. In: Hester RE, Harrison RM (Eds.), Air Pollution and Health. The Royal Society of Chemistry, pp. 33–56 10.1039/9781847550095-00033.

[R56] Lima ALC, Farrington JW, Reddy CM, 2005. Combustion-derived polycyclic aromatic hydrocarbons in the environment - a review. Environ. Forensic 6, 109–131. 10.1080/15275920590952739.

[R57] Liu G, Niu Z, Van Niekerk D, Xue J, Zheng L, 2008. Polycyclic aromatic hydrocarbons (PAHs) from coal combustion: emissions, analysis, and toxicology. Rev. Environ. Contam. Toxicol 10.1007/978-0-387-71724-1_1. 18020302

[R58] Liu C, Zhang P, Yang B, Wang Y, Shu J, 2012. Kinetic studies of heterogeneous reactions of polycyclic aromatic hydrocarbon aerosols with NO3 radicals. Environ. Sci. Technol 46, 7575–7580. 10.1021/es301403d. 22747347

[R59] Liu Y, Goodrick S, Heilman W, 2014. Wildland fire emissions, carbon, and climate: wildfire-climate interactions. For. Ecol. Manag 317, 80–96. 10.1016/j.foreco.2013.02.020.

[R60] Mandalakis M, Gustafsson Ö, Alsberg T, Egebäck AL, Reddy CM, Xu L, Klanova J, Holoubek I, Stephanou EG, 2005. Contribution of biomass burning to atmospheric polycyclic aromatic hydrocarbons at three European background sites. Environ. Sci. Technol 39, 2976–2982. 10.1021/es048184v. 15926541

[R61] Masclet P, Bresson MA, Mouvier G, 1987. Polycyclic aromatic hydrocarbons emitted by power stations, and influence of combustion conditions. Fuel 66, 556–562. 10.1016/0016-2361(87)90163-3.

[R62] Matteo B, Luca M, Federica P, Daniele CB, Marina C, 2019. Urban air pollution, climate change and wildfires: the case study of an extended forest fire episode in northern Italy favoured by drought and warm weather conditions. Energy Rep. 6, 781–786. 10.1016/j.egyr.2019.11.002.

[R63] Mazzoleni LR, Zielinska B, Moosmüller H, 2007. Emissions of levoglucosan, methoxy phenols, and organic acids from prescribed burns, laboratory combustion of wildland fuels, and residential wood combustion. Environ. Sci. Technol 41, 2115–2122. 10.1021/es061702c. 17438751

[R64] McMohan CK, Tsoukalas SN, 1987. Polynuclear aromatic hydrocarbons in forest fire smoke. Carcinogenesis 61–73.

[R65] Mesquita SR, van Drooge B, Barata C, Vieira N, Guimarães L, Piña B, 2014. Toxicity of atmospheric particle-bound PAHs: an environmental perspective. Environ. Sci. Pollut. Res 21, 11623–11633. 10.1007/s11356-014-2628-y. 24595747

[R66] Nayebzadeh M, Vahedpour M, 2017. A review on reactions of polycyclic aromatic hydrocarbons with the most abundant atmospheric chemical fragments: theoretical and experimental data. Prog. React. Kinet. Mech 42, 201–220. 10.3184/146867817X14821527549293.

[R67] Nicovich JM, Thompson RL, Ravishankara AR, 1981. Kinetics of the reactions of the hydroxyl radical with xyienes. J. Phys. Chem 85, 2913–2916. 10.1021/j150620a012.

[R68] Niu X, Chuang HC, Wang X, Ho SSH, Li L, Qu L, Chow JC, Watson JG, Sun J, Lee S, Cao J, Ho KF, 2020. Cytotoxicity of PM2.5 vehicular emissions in the Shing Mun Tunnel, Hong Kong. Environ. Pollut 263, 114386. 10.1016/j.envpol.2020.114386. 32203846

[R69] O’Dell K, Hornbrook RS, Permar W, Levin EJT, Garofalo LA, Apel EC, Blake NJ, Jarnot A, Pothier MA, Farmer DK, Hu L, Campos T, Ford B, Pierce JR, Fischer EV, 2020. Hazardous air pollutants in fresh and aged Western US wildfire smoke and implications for long-term exposure. Environ. Sci. Technol 54, 11838–11847. 10.1021/acs.est.0c04497. 32857515

[R70] Palm BB, Campuzano-Jost P, Ortega AM, Day DA, Kaser L, Jud W, Karl T, Hansel A, Hunter JF, Cross ES, Kroll JH, Peng Z, Brune WH, Jimenez JL, 2016. In situ secondary organic aerosol formation from ambient pine forest air using an oxidation flow reactor. Atmos. Chem. Phys 16, 2943–2970. 10.5194/acp-16-2943-2016.

[R71] Pankow JF, 1994. An absorption model of gas/particle partitioning of organic compounds in the atmosphere. Atmos. Environ 28, 185–188. 10.1016/1352-2310(94)90093-0.

[R72] Rajput P, Sarin MM, Rengarajan R, Singh D, 2011. Atmospheric polycyclic aromatic hydrocarbons (PAHs) from post-harvest biomass burning emissions in the Indo-Gangetic Plain: isomer ratios and temporal trends. Atmos. Environ 45, 6732–6740. 10.1016/j.atmosenv.2011.08.018.

[R73] Ramírez N, Cuadras A, Rovira E, Marcé RM, Borrull F, 2011. Risk assessment related to atmospheric polycyclic aromatic hydrocarbons in gas and particle phases near industrial sites. Environ. Health Perspect 119, 1110–1116. 10.1289/ehp.1002855. 21478082 PMC3237345

[R74] Reid CE, Brauer M, Johnston FH, Jerrett M, Balmes JR, Elliott CT, 2016. Health impacts of wildfire smoke. Environ. Health Perspect 124, 1334–1343. 10.1289/ehp.1409277. 27082891 PMC5010409

[R75] Reisen F, Arey J, 2005. Atmospheric reactions influence seasonal PAH and nitro-PAH concentrations in the Los Angeles basin. Environ. Sci. Technol 39, 64–73. 10.1021/es035454l. 15667076

[R76] Rogge WF, Hildemann LM, Mazurek MA, Cass GR, Simoneit BRT, 1993. Sources of fine organic aerosol. 2. Noncatalyst and catalyst-equipped automobiles and heavy-duty diesel trucks. Environ. Sci. Technol 27, 636–651. 10.1021/es00041a007.

[R77] Saffari A, Hasheminassab S, Wang D, Shafer MM, Schauer JJ, Sioutas C, 2015. Impact of primary and secondary organic sources on the oxidative potential of quasi-ultrafine particles (PM0.25) at three contrasting locations in the Los Angeles Basin. Atmos. Environ 120, 286–296. 10.1016/j.atmosenv.2015.09.022.

[R78] Samburova V, Connolly J, Gyawali M, Yatavelli RLN, Watts AC, Chakrabarty RK, Zielinska B, Moosmüller H, Khlystov A, 2016a. Polycyclic aromatic hydrocarbons in biomass-burning emissions and their contribution to light absorption and aerosol toxicity. Sci. Total Environ 568, 391–401. 10.1016/j.scitotenv.2016.06.026. 27304373

[R79] Samburova V, Zielinska B, Khlystov A, 2017. Do 16 polycyclic aromatic hydrocarbons represent PAH air toxicity? Toxics 5, 29–33. 10.3390/toxics5030017. 29051449 PMC5634701

[R80] Sasaki J, Aschmann SM, Kwok ESC, Atkinson R, Arey J, 1998. Products of the gas-phase OH and NO3 radical-initiated reactions of naphthalene. Environ. Sci. Technol 31, 3173–3179. 10.1021/es9701523.

[R81] Schauer JJ, Kleeman MJ, Cass GR, Simoneit BRT, 2001a. Measurement of emissions from air pollution sources. 3. C1–C29 organic compounds from fireplace combustion of wood. Environ. Sci. Technol 35, 1716–1728. 10.1021/es001331e. 11355184

[R82] Sengupta D, Samburova V, Bhattarai C, Watts AC, Moosmüller H, Khlystov AY, 2020. Polar semivolatile organic compounds in biomass-burning emissions and their chemical transformations during aging in an oxidation flow reactor. Atmos. Chem. Phys 20 (13), 8227–8250.

[R83] Shen G, Wang W, Yang Y, Ding J, Xue M, Min Y, Zhu C, Shen H, Li W, Wang B, Wang R, Wang X, Tao S, Russell AG, 2011. Emissions of PAHs from indoor crop residue burning in a typical rural stove: emission factors, size distributions, and gas-particle partitioning. Environ. Sci. Technol 45, 1206–1212. 10.1021/es102151w. 21247097 PMC3092001

[R84] Shen G, Tao S, Wei S, Zhang Y, Wang R, Wang B, Li W, Shen H, Huang Y, Chen Y, Chen H, Yang Y, Wang W, Wei W, Wang Xilong, Liu W, Wang Xuejun, Simonich SLM, 2012. Reductions in emissions of carbonaceous particulate matter and polycyclic aromatic hydrocarbons from combustion of biomass pellets in comparison with raw fuel burning. Environ. Sci. Technol 46, 6409–6416. 10.1021/es300369d. 22568759 PMC3377013

[R85] Shen H, Huang Y, Wang R, Zhu D, Li W, Shen G, Wang B, Zhang Y, Chen Y, Lu Y, Chen H, Li T, Sun K, Li B, Liu W, Liu J, Tao S, 2013. Global atmospheric emissions of polycyclic aromatic hydrocarbons from 1960 to 2008 and future predictions. Environ. Sci. Technol 47, 6415–6424. 10.1021/es400857z. 23659377 PMC3753807

[R86] Shen H, Tao S, Liu J, Huang Y, Chen H, Li W, Zhang Y, Chen Y, Su S, Lin N, Xu Y, Li B, Wang X, Liu W, 2014. Global lung cancer risk from PAH exposure highly depends on emission sources and individual susceptibility. Sci. Rep 4, 1–8. 10.1038/srep06561. PMC4190535 25297709

[R87] Shen G, Preston W, Ebersviller SM, Williams C, Faircloth JW, Jetter JJ, Hays MD, 2017. Polycyclic aromatic hydrocarbons in fine particulate matter emitted from burning kerosene, liquid petroleum gas, and wood fuels in household cookstoves. Energy Fuels 31, 3081–3090. 10.1021/acs.energyfuels.6b02641. 30245546 PMC6145494

[R88] Shrivastava M, Lou S, Zelenyuk A, Easter RC, Corley RA, Thrall BD, Rasch PJ, Fast JD, Simonich SLM, Shen H, Tao S, 2017. Global long-range transport and lung cancer risk from polycyclic aromatic hydrocarbons shielded by coatings of organic aerosol. Proc. Natl. Acad. Sci. U. S. A 114, 1246–1251. 10.1073/pnas.1618475114. 28115713 PMC5307436

[R89] Sigsgaard T, Forsberg B, Annesi-Maesano I, Blomberg A, Bølling A, Boman C, Bønløkke J, Brauer M, Bruce N, Héroux ME, Hirvonen MR, Kelly F, Künzli N, Lundbäck B, Moshammer H, Noonan C, Pagels J, Sallsten G, Sculier JP, Brunekreef B, 2015. Health impacts of anthropogenic biomass burning in the developed world. Eur. Respir. J 46, 1577–1588. 10.1183/13993003.01865-2014. 26405285

[R90] Simoneit BRT, 2002. Biomass burning — a review of organic tracers for smoke from incomplete combustion. Appl. Geochem 17, 129–162. 10.1016/S0883-2927(01)00061-0.

[R91] Sofowote UM, Hung H, Rastogi AK, Westgate JN, Su Y, Sverko E, D’Sa I, Roach P, Fellin P, McCarry BE, 2010. The gas/particle partitioning of polycyclic aromatic hydrocarbons collected at a sub-Arctic site in Canada. Atmos. Environ 44, 4919–4926. 10.1016/j.atmosenv.2010.08.028.

[R92] Sofowote UM, Hung H, Rastogi AK, Westgate JN, Deluca PF, Su Y, McCarry BE, 2011. Assessing the long-range transport of PAH to a sub-Arctic site using positive matrix factorization and potential source contribution function. Atmos. Environ 45, 967–976. 10.1016/j.atmosenv.2010.11.005.

[R93] Stein SE, Fahr A, 1985. High-temperature stabilities of hydrocarbons. J. Phys. Chem 89, 3714–3725. 10.1021/j100263a027.

[R94] Stephens SL, Burrows N, Buyantuyev A, Gray RW, Keane RE, Kubian R, Liu S, Seijo F, Shu L, Tolhurst KG, Van Wagtendonk JW, 2014. Temperate and boreal forest mega-fires: characteristics and challenges. Front. Ecol. Environ 12, 115–122. 10.1890/120332.

[R95] Sun J, Shen Z, Zeng Y, Niu X, Wang J, Cao J, Gong X, Xu H, Wang T, Liu H, Yang L, 2018. Characterization and cytotoxicity of PAHs in PM2.5 emitted from residential solid fuel burning in the Guanzhong Plain, China. Environ. Pollut 241, 359–368. 10.1016/j.envpol.2018.05.076. 29852439

[R96] Tian J, Chow JC, Cao J, Han Y, Ni H, Chen LWA, … Watson JG, 2015. A biomass combustion chamber: Design, evaluation, and a case study of wheat straw combustion emission tests. Aerosol Air Qual. Res 15 (5), 2104–2114.

[R97] U. S. Food and Drug Administration/Center for Biologics Evaluation and Research, 1995. Guideline for Industry: Text on Validation of Analytical Procedures (ICH-Q2A). Guidelines 9.

[R98] USEPA, 1999. Compendium of Methods for the Determination of Toxic Organic Compounds in Ambient Air, Compendium Method TO-13A: Determination of Polycyclic Aromatic Hydrocarbons (PAHs) in Ambient Air Using Gas Chromatography/Mass Spectrometry (GC/MS). 78. Cent. Environ. Res. Inf. Off. Res. Dev. U.S. Environ. Prot. Agency, Cincinnati, OH 45268 II.

[R99] Vachula RS, Karp AT, Denis EH, Balascio NL, Canuel EA, Huang Y, 2022. Spatially calibrating polycyclic aromatic hydrocarbons (PAHs) as proxies of area burned by vegetation fires: insights from comparisons of historical data and sedimentary PAH fluxes. Palaeogeogr. Palaeoclimatol. Palaeoecol 596, 110995. 10.1016/j.palaeo.2022.110995.

[R100] Vakkari V, Beukes JP, Dal Maso M, Aurela M, Josipovic M, van Zyl PG, 2018. Major secondary aerosol formation in southern African open biomass burning plumes. Nat. Geosci 11, 580–583. 10.1038/s41561-018-0170-0.

[R101] Visentin M, Pagnoni A, Sarti E, Pietrogrande MC, 2016. Urban PM2.5 oxidative potential: importance of chemical species and comparison of two spectrophotometric cell-free assays. Environ. Pollut 219, 72–79. 10.1016/j.envpol.2016.09.047. 27661730

[R102] Wang F, Lin T, Li Y, Ji T, Ma C, Guo Z, 2014. Sources of polycyclic aromatic hydrocarbons in PM2.5 over the East China Sea, a downwind domain of East Asian continental outflow. Atmos. Environ 92, 484–492. 10.1016/j.atmosenv.2014.05.003.

[R103] Watts AC, Samburova V, 2020. Criteria-based identification of important fuels for wildland fire emission research. Atmosphere 11, 640. 10.3390/atmos11060640.

[R104] Wei C, Han Y, Bandowe BAM, Cao J, Huang RJ, Ni H, Tian J, Wilcke W, 2015. Occurrence, gas/particle partitioning and carcinogenic risk of polycyclic aromatic hydrocarbons and their oxygen and nitrogen containing derivatives in Xi'an, central China. Sci. Total Environ 505, 814–822. 10.1016/j.scitotenv.2014.10.054. 25461084

[R105] Wentworth GR, Aklilu Y.abeba, Landis MS, Hsu YM, 2018. Impacts of a large boreal wildfire on ground level atmospheric concentrations of PAHs, VOCs and ozone. Atmos. Environ 178, 19–30. 10.1016/j.atmosenv.2018.01.013. PMC5906807 29681759

[R106] Westerling AL, Bryant BP, Preisler HK, Holmes TP, Hidalgo HG, Das T, Shrestha SR, 2011. Climate change and growth scenarios for California wildfire. Clim. Chang 109, 445–463. 10.1007/s10584-011-0329-9.

[R107] Wong JPS, Tsagkaraki M, Tsiodra I, Mihalopoulos N, Violaki K, Kanakidou M, Sciare J, Nenes A, Weber RJ, 2019. Effects of atmospheric processing on the oxidative potential of biomass burning organic aerosols. Environ. Sci. Technol 53, 6747–6756. 10.1021/acs.est.9b01034. 31091086

[R108] Wu D, Zheng H, Li Q, Jin L, Lyu R, Ding X, Huo Y, Zhao B, Jiang J, Chen J, Li X, Wang S, 2022. Toxic potency-adjusted control of air pollution for solid fuel combustion. Nat. Energy 7, 194–202. 10.1038/s41560-021-00951-1.

[R109] Yuan H, Tao S, Li B, Lang C, Cao J, Coveney RM, 2008. Emission and outflow of polycyclic aromatic hydrocarbons from wildfires in China. Atmos. Environ 42, 6828–6835. 10.1016/j.atmosenv.2008.05.033.

[R110] Zelenyuk A, Imre DG, Wilson J, Bell DM, Suski KJ, Shrivastava M, Beránek J, Alexander ML, Kramer AL, Massey Simonich SL, 2017. The effect of gas-phase polycyclic aromatic hydrocarbons on the formation and properties of biogenic secondary organic aerosol particles. Faraday Discuss. 200, 143–164. 10.1039/c7fd00032d. 28581016 PMC9918307

[R111] Zeng M, Liao Z, Wang L, 2020. Atmospheric oxidation of gaseous anthracene and phenanthrene initiated by OH radicals. Atmos. Environ 234, 117587. 10.1016/j.atmosenv.2020.117587.

[R112] Zhang J, Li J, Wang P, Chen G, Mendola P, Sherman S, Ying Q, 2017. Estimating population exposure to ambient polycyclic aromatic hydrocarbon in the United States – part I: model development and evaluation. Environ. Int 99, 263–274. 10.1016/j.envint.2016.12.002. 27988136 PMC6198650

[R113] Zhao N, Zhang Q, Wang W, 2016. Atmospheric oxidation of phenanthrene initiated by OH radicals in the presence of O2 and NOx — a theoretical study. Sci. Total Environ 563–564, 1008–1015. 10.1016/j.scitotenv.2016.01.089. 27169729

[R114] Zhou S, Wenger JC, 2013. Kinetics and products of the gas-phase reactions of acenaphthylene with hydroxyl radicals, nitrate radicals and ozone. Atmos. Environ 75, 103–112. 10.1016/j.atmosenv.2013.04.049.

[R115] Zhou S, Hwang BCH, Lakey PSJ, Zuend A, Abbatt JPD, Shiraiwa M, 2019. Multiphase reactivity of polycyclic aromatic hydrocarbons is driven by phase separation and diffusion limitations. Proc. Natl. Acad. Sci 201902517. 10.1073/pnas.1902517116. PMC6575172 31142653

[R116] Zielinska B, Sagebiel J, Arnott WP, Rogers CF, Kelly KE, Wagner DA, Lighty JS, Sarofim AF, Palmer G, 2004. Phase and size distribution of polycyclic aromatic hydrocarbons in diesel and gasoline vehicle emissions. Environ. Sci. Technol 38, 2557–2567. 10.1021/es030518d. 15180051

