# Supplementary material for: Emission factors for polycyclic aromatic hydrocarbons from laboratory biomass-burning and their chemical transformations during aging in an oxidation flow reactor
Source: Sci Total Environ. Author manuscript; Available in PMC 2024 Apr 3. (PMC10990481; doi:10.1016/j.scitotenv.2023.161857)
Supplement: Supplementary material [file NIHMS1978398-supplement-Supplementary_material.docx]

*Supplementary material*

**Emission factors for polycyclic aromatic hydrocarbons from laboratory biomass-burning and their chemical transformations during aging in an oxidation flow reactor**

Deep Sengupta [^a^](#_bookmark0)^,b^[⁎](#_bookmark3), Vera Samburova [^a^](#_bookmark0) , Chiranjivi Bhattarai [^a^](#_bookmark0), Hans Moosmüller [^a^](#_bookmark0), Andrey Khlystov [^a^](#_bookmark0)

[^a^](#_bookmark0)Desert Research Institute, Reno, Nevada, USA

^b^University of California, Berkeley, California, USA

^*^Corresponding author


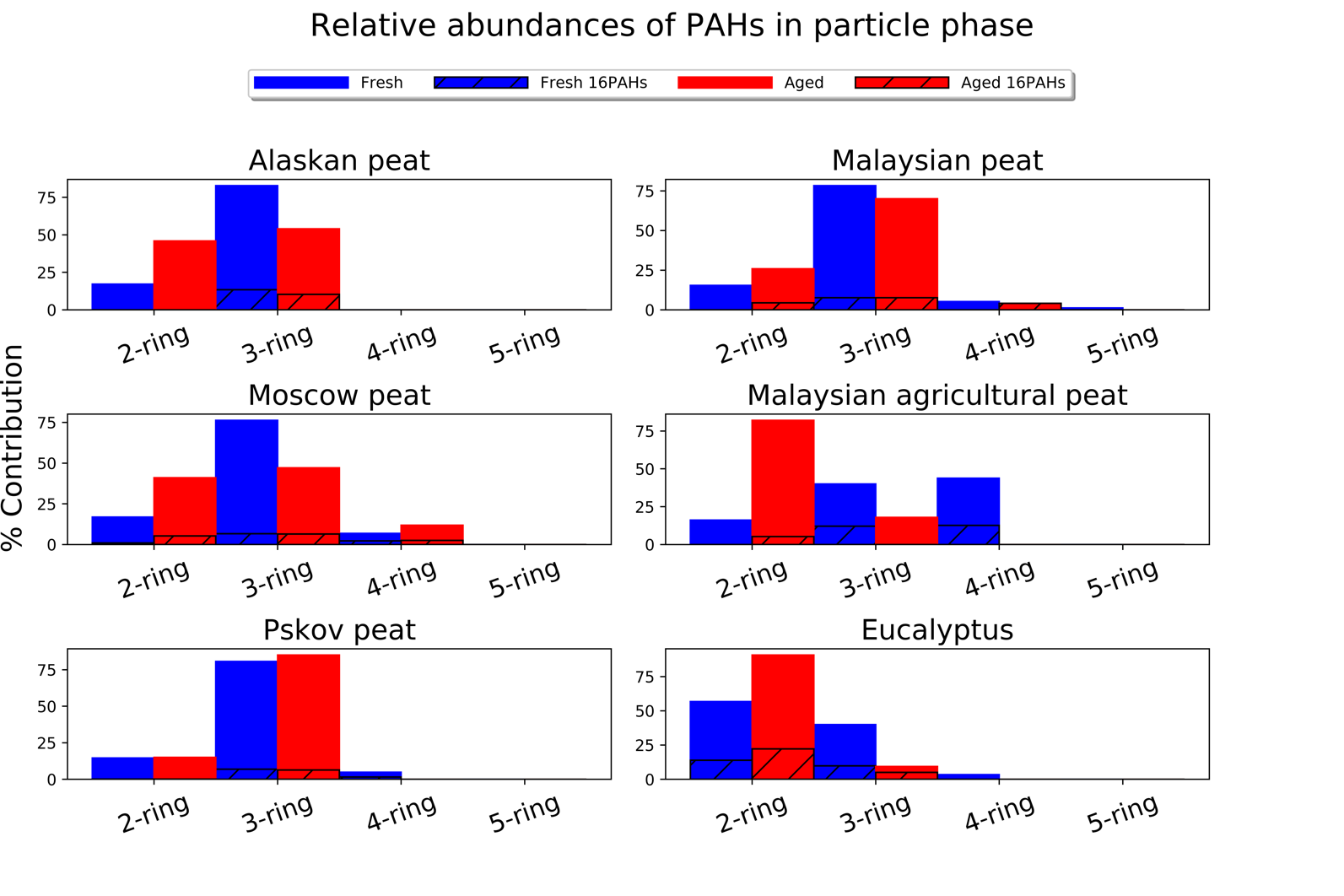


**Figure S1**. Fractional contribution of PAHs with different number of aromatic rings towards total particulate matter mass emissions for fresh and OFR-aged aerosols from combustion of six different fuels: Alaskan peat, Malaysian peat, Moscow peat, Malaysian agricultural peat, Pskov peat, Eucalyptus.

**
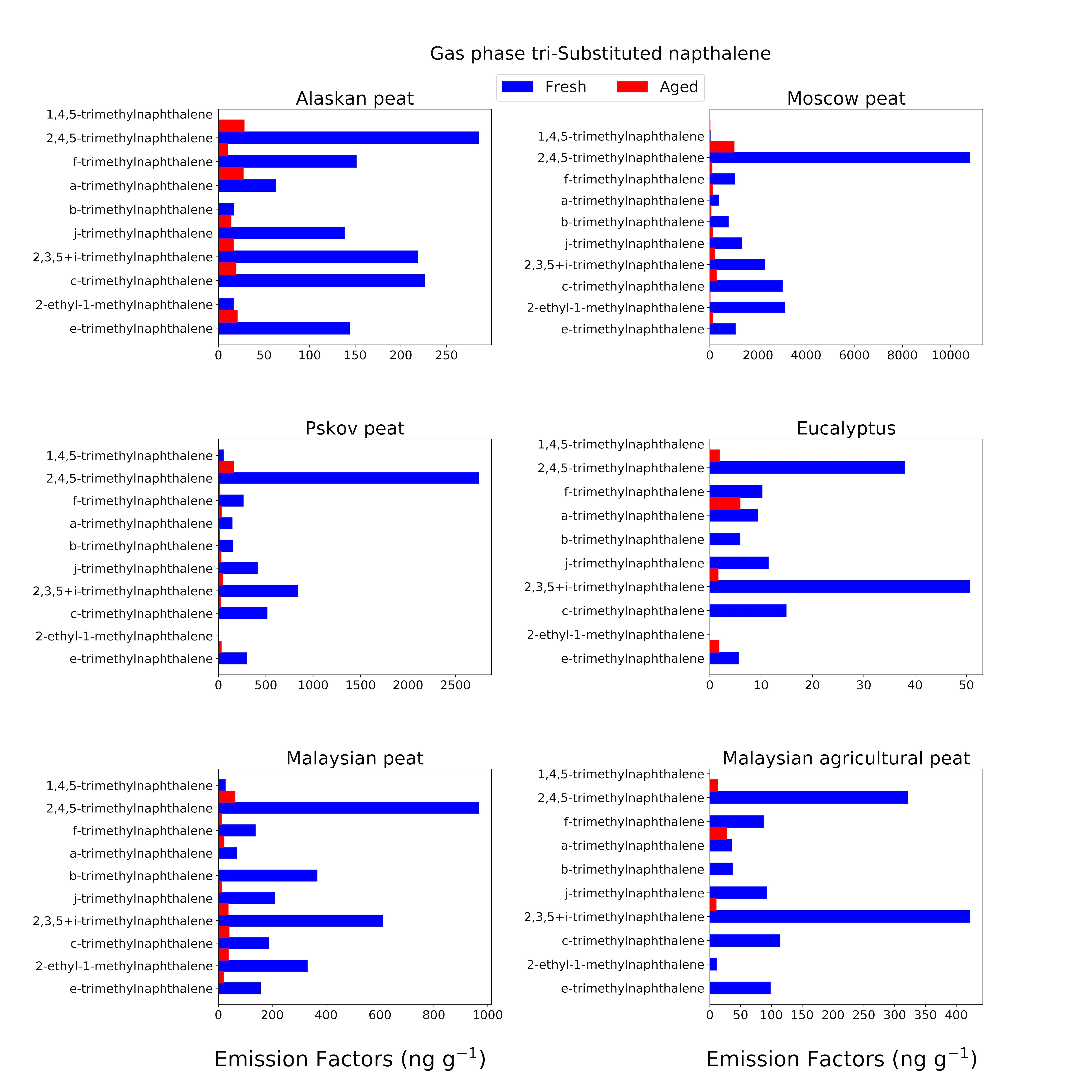
**

**Figure S2**. EFs for tri-substituted naphthalenes in gas-phase from fresh and OFR-aged BB emissions for six different fuels. We did not burn fuels in replicates, and standard deviations (SD) were calculated based on replicate analysis of BB emissions from similar fuels (with identical experimental conditions) during our previous BB campaigns (Samburova et al., 2016) where SD ranged between 45 and 50% for tri-substituted napthalenes.


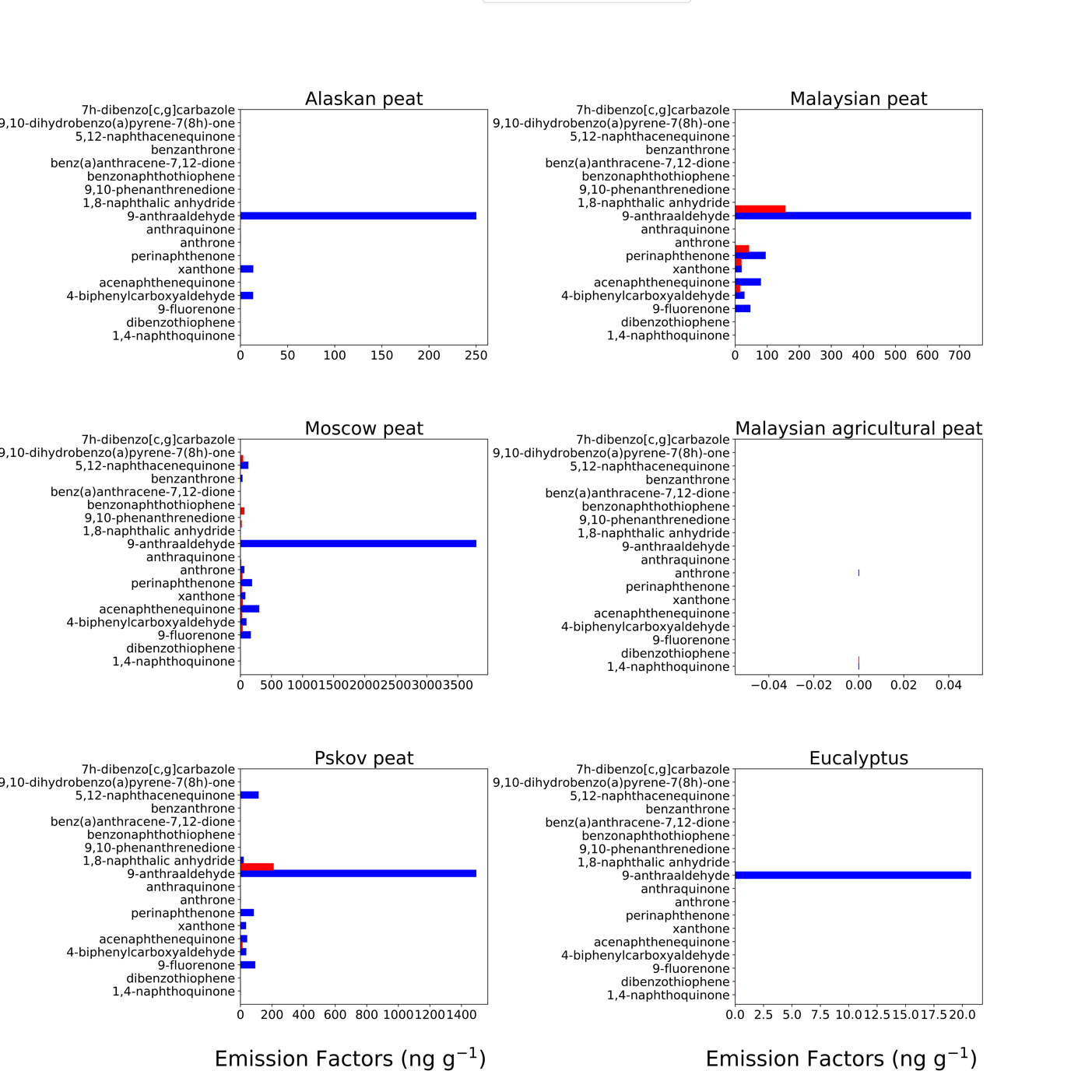


**Figure S3**. EFs for oxo-PAHs in particle-phase for fresh and OFR-aged BB emissions from six different fuels.


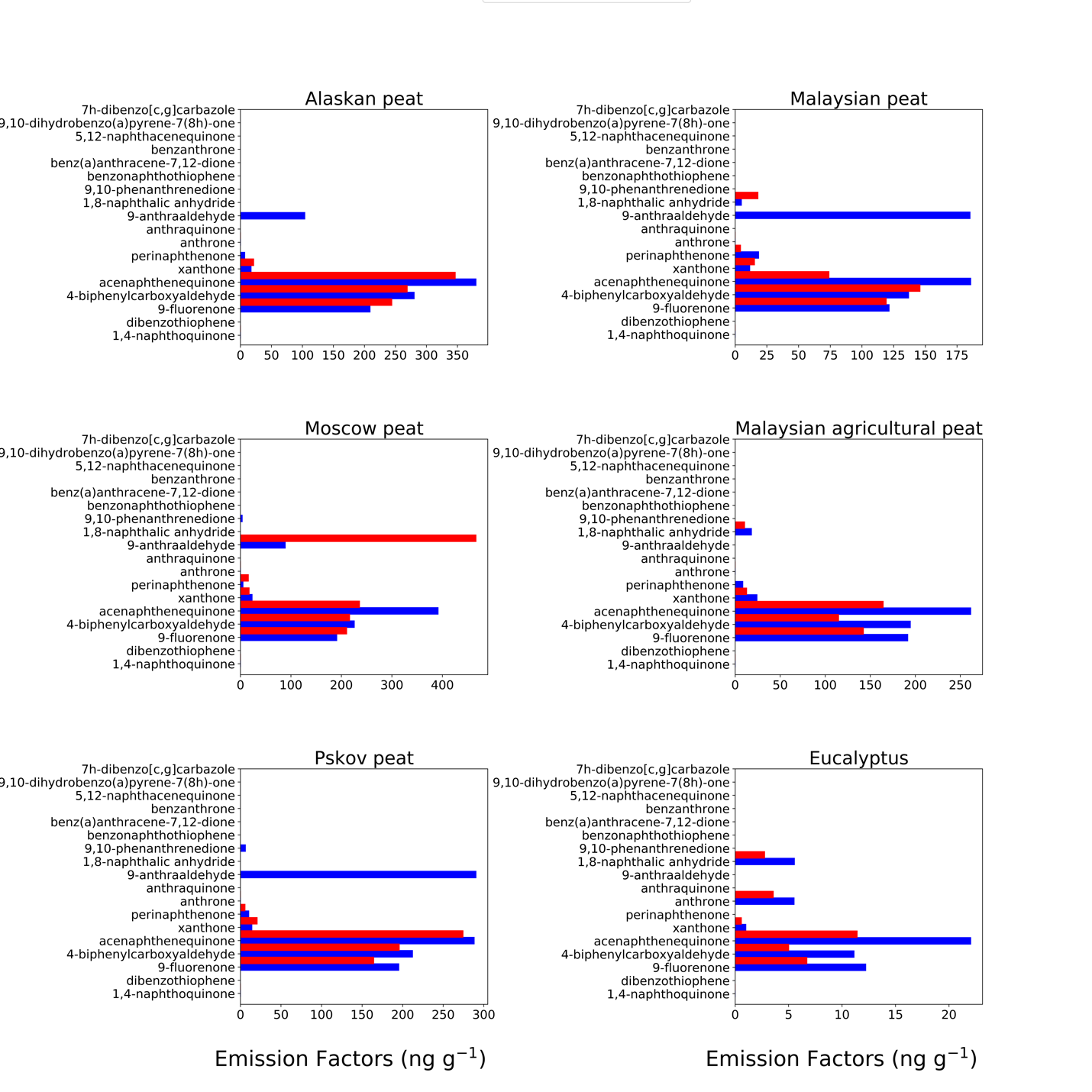


**Figure S4**. EFs for gas-phase oxo-PAHs for fresh and OFR-aged BB emissions from six different fuels.

**

**

**Figure S5**. EFs for mono-substituted phenanthrenes BB emissions for six different fuel type presented separately for gas- (open bars) and particle- (solid bars) phase species and for fresh (blue) and OFR-aged (red) emissions. Standard deviations of the EFs were calculated based on replicated burns performed by Samburova et al. (2016a) and they ranged between 58% and 53% for substituted phenanthrenes and anthracenes respectively (not shown in figure) .


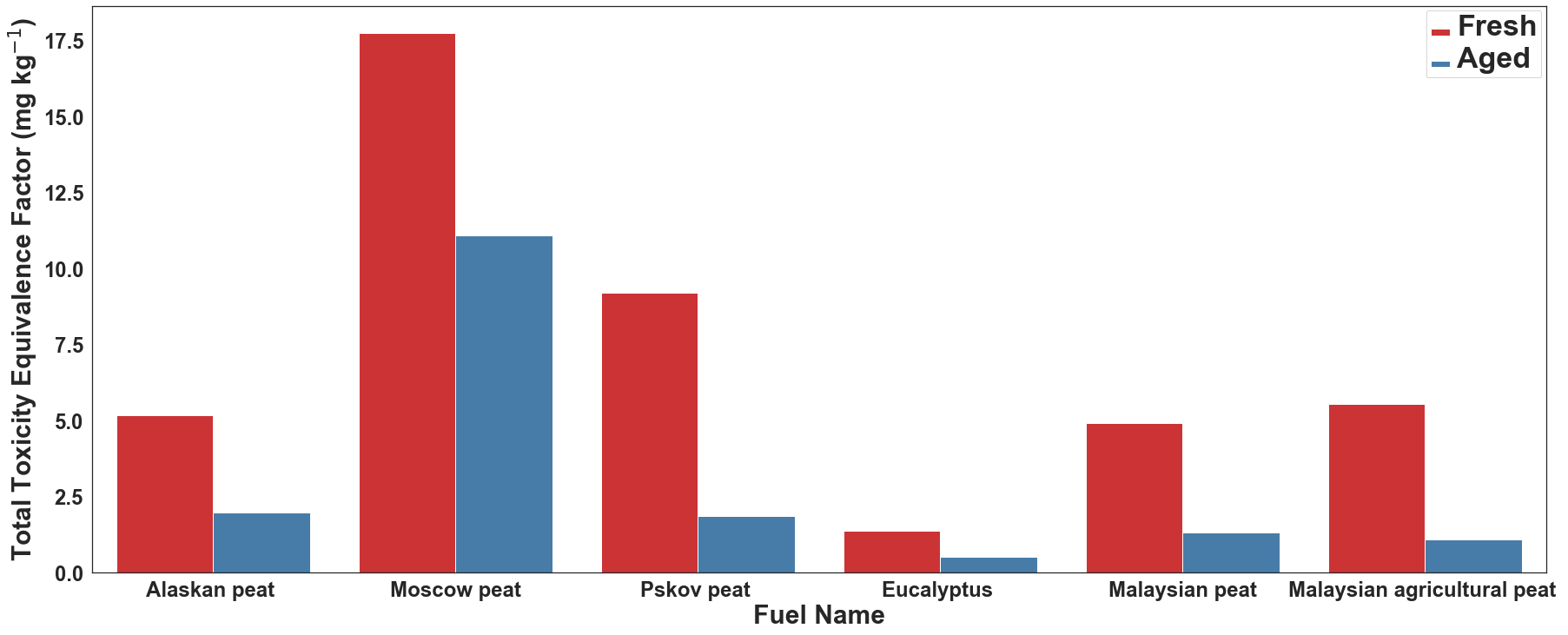


**Figure S6**. Changes in Toxicity Equivalence Factors (TEFs) between fresh and OFR-aged BB emissions from six important biomass fuels.

**Table S1**: Fuel-based emission factors (EFs) of 2 – 5 ring PAHs for combustion of six fuels (in µg g^−1^): Alaskan peat, Malaysian peat, Moscow peat, Malaysian agricultural peat, Pskov peat, and eucalyptus

**Table S2**: Substituted and Unsubstituted Naphthalene in gas phase (in ng g^−1^):

**Table S3**: Unsubstituted PAHs in both gas and particle phase and their fate after OFR aging (in ng g^−1^):

Total Toxic Equivalence Factor = Total_TEF

$Total\_TEF=\sum{EF}_{i}*TEF_{i}$_______________________________Equation.1.

$EF_{i}$ = emission factors for individual species in either gas phase, particle phase or (gas + particle) phase

$TEF_{i}$ values are presented in Table S4 bellow

**Table S4**: TEF values from available literature (Nisbet and LaGoy, 1992) and nearest neighbor assignment.

**References:**

Nisbet, I.C.T., LaGoy, P.K., 1992. Toxic equivalency factors (TEFs) for polycyclic aromatic hydrocarbons (PAHs). Regul. Toxicol. Pharmacol. 16, 290–300. https://doi.org/10.1016/0273-2300(92)90009-X

Samburova, V., Connolly, J., Gyawali, M., Yatavelli, R.L.N., Watts, A.C., Chakrabarty, R.K., Zielinska, B., Moosmüller, H., Khlystov, A., 2016. Polycyclic aromatic hydrocarbons in biomass-burning emissions and their contribution to light absorption and aerosol toxicity. Sci. Total Environ. 568, 391–401. https://doi.org/10.1016/j.scitotenv.2016.06.026
